# Emergency Department Utilization by Women of Reproductive Age for Mental Illness in St. Louis Before and During the COVID-19 Pandemic

**DOI:** 10.3390/ijerph23020177

**Published:** 2026-01-30

**Authors:** Jen Jen Chang, Christopher D. Hopwood, Yuki Sugawara, Abigail Andresen, Thomas E. Burroughs, Aya Bou Fakhreddine, Steven E. Rigdon

**Affiliations:** 1 Department of Epidemiology and Biostatistics, College for Public Health and Social Justice, Saint Louis University, St. Louis, MO 63103, USA; jenjen.chang@slu.edu (J.J.C.); chris.hopwood@slu.edu (C.D.H.); yuki.sugawara@slu.edu (Y.S.); aya.boufakhreddine@slu.edu (A.B.F.); 2 City of St. Louis Department of Public Health, St. Louis, MO 63134, USA; 3 Department of Health Management and Policy, College for Public Health and Social Justice, Saint Louis University, St. Louis, MO 63103, USA; tom.burroughs@slu.edu

**Keywords:** mental illness, emergency department visit, CAR model, hierarchical Bayesian model, random walk, space–time interaction

## Abstract

**Highlights:**

**Public health relevance—How does this work relate to a public health issue?**

- Women are generally at greater risk of mental illness than men
- Urban residents may increasingly rely on emergency departments for acute care due to limited capacity

**Public health significance—Why is this work of significance to public health?**

- Mental health among women of child-bearing age affects not just the mother, but also the child.
- Geospatial tools reveal greatest service gaps among reproductive-age women with behavioral health conditions, directing interventions to unmet needs.

**Public health implications—What are the key implications or messages for practitioners, policy makers and/or researchers in public health?**

- The spatio-temporal model indicates that census tracts with a higher proportion of public insurance, a higher proportion of those with a high school diploma, and greater age tended to have a higher demand for emergency mental health resources.
- Those census tracts with a higher proportion of private insurance and higher income tended to have a lower demand for mental health resources.
- The analysis captures ED utilization’s temporal trends and neighborhood variations, providing actionable insights for data-driven strategies that target high-need areas.

**Abstract:**

Mental illness and related health inequities are disproportionately concentrated in economically disadvantaged urban neighborhoods. The COVID-19 pandemic has been associated with a rise in mental illness prevalence, with women generally at greater risk than men. Urban areas facing multiple structural and socioeconomic challenges may have limited capacity to meet the mental healthcare needs of residents, leading to increased reliance on emergency departments (EDs) for acute care. This ecological study uses data over four years (2018–2021) and examines spatial variations in ED utilization at the census tract level, focusing on geographic areas with women of reproductive age diagnosed with mental illness to compare patterns before and during the COVID-19 pandemic. Of the 22,565 ED visits in the four-year period, 12,832 occurred before COVID-19 and 9733 during COVID-19. Our findings highlight persistent structural disparities in mental healthcare access across census tracts characterized by high concentrations of vulnerable women of reproductive age. Understanding these spatial disparities allows for geographically targeted interventions and the prioritization of resources for neighborhoods identified as most underserved.

## 1. Mental Health Among Women of Reproductive Age

Mental illness among women of reproductive age represents a significant public health burden in both the United States and globally. In the U.S., approximately 20% of women in this demographic experience psychological distress, with depression and anxiety affecting 7–14% and 12–15%, respectively [1,2]. These prevalence rates rank among the highest compared to other high-income countries [3]. The associated financial burden is substantial, with the annual economic cost of major depressive disorder (MDD) in adults estimated at USD 326.2 billion, largely driven by healthcare expenditures, productivity losses, and suicide-related costs [4]. Mental health conditions in women of reproductive age often co-occur with chronic diseases such as hypertension, obesity, and diabetes, amplifying both individual and societal burdens [5,6]. In addition to affecting educational and occupational functioning, poor mental health can strain social and family relationships and reduce workplace productivity [1,7].

Critically, mental health conditions during the reproductive years have also been associated with adverse health outcomes across the life course, including during pregnancy and postpartum, as well as outside of reproductive events [5,8,9]. Women of childbearing age face increased vulnerability to mental health challenges, including depression, anxiety, and chronic stress. These conditions may be intensified by hormonal changes, nutrition, thyroid dysfunction, limited social support, and adverse life events. These issues span the entire reproductive life course and carry significant implications for physical, emotional, and reproductive health. Approximately 12.8% of all hospitalizations among women of reproductive age are related to mental health or substance use disorders, with nearly half involving both concurrently [10]. Substance use disorder (SUD) alone affects an estimated 9.7% of women in this age group [11], and those with SUD are more likely to utilize social and mental health services than their peers without SUD [1]. Although the co-occurrence of depression and anxiety is common, fewer than 25% of women with both SUD and mental health conditions receive behavioral health treatment [2].

The COVID-19 pandemic significantly intensified this public health burden, causing disproportionate disruptions in mental healthcare for women in the target population. Heightened stress, social isolation, and economic instability during the pandemic led to increased prevalence and severity of conditions such as anxiety and depression [12]. Specifically, studies on perinatal mental health indicate an increase in perinatal depression symptoms related to pandemic conditions. Telehealth and virtual visits became rapidly integrated into patient care, offering temporary and isolated solutions to mental health services. Although this shift benefited many individuals, it also created intervention-generated inequalities in access to care for marginalized communities. Lack of digital access, digital health literacy, and financial costs associated with telehealth medicine could be a contributing factor to higher reliance on emergency department use for acute mental health crises. The transition to telehealth systematically created tiered systems of care: high-quality, comprehensive video medical visits for some and limited, inadequate audio-only care for others.

Emergency departments (EDs) often serve as the primary access point for medical care. This role became even more pronounced during the COVID-19 pandemic, as service disruptions and increased stress exacerbated medical and/or behavioral health needs. Female sex has been associated with higher odds of ED utilization, with women accounting for a large proportion of mental health-related ED visits [13,14]. Among women of childbearing age, frequent ED use reflects not only acute medical needs or substance-related crises but also long-standing structural barriers to timely and comprehensive medical care. Females are more likely to rely on EDs due to cost-related barriers, gaps in insurance coverage, caregiving responsibilities, and difficulty accessing timely outpatient and preventive services [14]. For example, women insured through Medicaid or other public insurance have substantially higher odds of ED use compared with those with private insurance, reflecting persistent financial and structural barriers to outpatient care. Caregiving responsibilities and logistical constraints, especially childcare needs, transportation difficulties, and lack of paid time off, further limit women’s ability to attend routine medical visits [15]. These barriers are more common among low-income women and women from racially and ethnically marginalized communities [15].

From 2006 to 2013, ED visits for depression, anxiety, and stress among women aged 15 years and older increased by 54.9%, reaching nearly 5000 visits per 100,000 women [16]. Together, these barriers support the need to consider gender and social context when examining ED utilization patterns among reproductive-age women. Among reproductive-age women, the high frequency of ED visits for medical and/or behavioral health concerns signals both individual distress and systemic failures in mental health prevention and service delivery [5,17,18].

### 1.1. Challenges in the City of St. Louis

The City of St. Louis faces profound and long-standing health inequities rooted in systemic racism, historical disinvestment, and unequal access to healthcare and resources. These long-standing issues have led to high rates of mental health and substance use challenges, particularly for women of childbearing age. This creates a constant, heavy burden on the city’s emergency rooms and healthcare systems.

**Figure 1:**
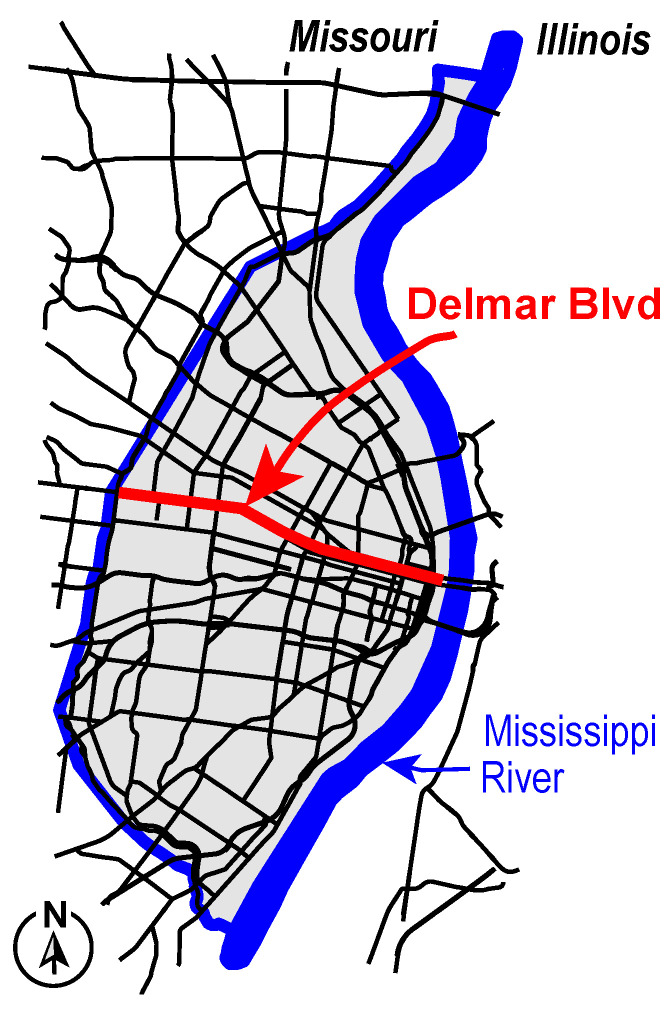
Delmar Blvd. divides the City of St. Louis into regions that are mostly African American (north) and mostly White (south).

Geographic disparities within the city are clearly illustrated through spatial data analysis. This reveals dramatic contrasts between northern and southern neighborhoods, divided by Delmar Boulevard, often referred to as the “Delmar Divide.” See Figure 1. North City neighborhoods, which are approximately 98% Black, were historically subjected to federal redlining policies in the 1930s that designated many of these communities as high-risk “red zones,” limiting access to mortgage financing and reinforcing patterns of disinvestment. In contrast, southern regions of the city, approximately 70% White, generally retained better access to financial resources and infrastructure investment. These historical patterns persist today, with neighborhoods north of Delmar experiencing higher rates of poverty, limited access to healthcare, and under-resourced public services, while southern neighborhoods maintain higher household incomes, stronger tax bases, and greater access to educational and healthcare infrastructure. Mental illness rates are highest in the City of St. Louis compared to St. Louis County and the state of Missouri across all racial groups, with disproportionately higher rates among Black residents [19]. Black individuals experience higher prevalence of anxiety disorders, mood disorders, schizophrenia, and co-occurring substance use disorders than White individuals [19]. Despite a relatively high concentration of mental health services, the City of St. Louis consistently reports elevated rates of mental health-related emergency department visits across all age groups, suggesting potential disparity in the ER and clinical services use related to mental health conditions [20]. Recent trends further indicate that females experience substantially higher emergency department visits for suicide attempts and suicidal ideation than males, with the highest rates observed among individuals aged 18–44 years, an age range including much of the reproductive life course [20]. This highlights the importance of examining neighborhood-level variation in mental health distress and emergency care utilization.

### 1.2. Geospatial Analysis to Guide Public Health Decision-Making

Geospatial analysis plays an increasingly vital role in public health and emergency response by identifying spatial patterns in health events and service utilization. These methods enable timely, targeted interventions and efficient resource deployment, particularly in urban settings where disparities in healthcare access are concentrated [21]. For reproductive-age women with behavioral health conditions, geospatial tools reveal where service gaps are heightened and guide interventions to areas of highest and unmet needs. In rapidly evolving contexts such as the COVID-19 pandemic, the ability to monitor where and when emergency services are most utilized becomes essential for equitable and effective public health response and outreach. The pandemic heightened behavioral health needs, disrupted outpatient care, and intensified caregiving burdens—especially for women with limited resources. In this context, location-based analysis became critical to track ED use and inform responsive care strategies.

Spatial modeling tools help identify disease clusters and vulnerable populations [22,23,24], while mobile mapping platforms support real-time data collection and decision-making in resource-constrained settings [22,25]. These systems transform routine data monitoring into evidence-based intervention areas, revealing priority census tracts, which are then used to create behavioral health strategies that are equitable, culturally appropriate, and responsive to community needs [26,27].

In the City of St. Louis, the burden of mental illness among reproductive-age women reflects national trends but is intensified by local structural inequities. The city’s 20% poverty rate, high chronic disease burden, and limited outpatient mental health infrastructure contribute to elevated ED use among women, particularly those aged 25 to 64 years [28,29]. Although women experience higher rates of mood and anxiety disorders than men, they often face greater barriers to care, including cost, stigma, and lack of access [28]. While perinatal mental health has been widely studied, less is known about behavioral health needs among reproductive-age women outside the context of pregnancy [1]. Although a growing body of neighborhood-level health equity research has documented spatial disparities in health outcomes and healthcare utilization, few studies examine intra-urban geospatial disparities in ED use, despite the influence of neighborhood-level conditions like poverty and segregation [30,31]. This gap is particularly relevant in St. Louis, where such disparities are both pronounced and underexplored.

While geospatial methods have been extensively applied to study infectious disease outbreaks [32,33] and chronic conditions affecting older populations [34,35], their use in examining mental health service utilization among reproductive-age women within urban neighborhoods remains limited [36]. This represents a critical gap, as reproductive-age women face unique mental health vulnerabilities that intersect with neighborhood-level social determinants, yet these spatial patterns are rarely examined with the granularity needed to inform targeted interventions. This study extends geospatial epidemiology into an underexplored domain by applying advanced spatio-temporal modeling to mental health-related ED visits among women aged 15–49. Set within a city characterized by structural racism and residential segregation, the analysis demonstrates how place-based research can elucidate disparities affecting vulnerable populations during public health crises.

To address these issues, we conducted an ecological, census-tract-level analysis of mental health-related emergency department utilization among women of reproductive age in the City of St. Louis from 2018 to 2021. While perinatal mental health has been widely studied, reproductive-age women outside of pregnancy represent an understudied population facing similar structural barriers. This study examines the full reproductive-age spectrum to capture mental health service utilization patterns that reflect broader healthcare access inequities affecting women of childbearing potential, recognizing that mental health needs and barriers to care extend well beyond pregnancy-specific contexts. We examined spatial and temporal patterns before and during the COVID-19 pandemic and applied a series of hierarchical spatio-temporal models to evaluate how different modeling approaches capture neighborhood-level variation and time-dependent change. Together, these analyses aim to inform public health surveillance and support geographically targeted behavioral health interventions in urban settings characterized by structural inequities.

## 2. Methods

### 2.1. Study Design and Data Source

This study examines geospatial differences in emergency department (ED) visits among reproductive-age women with mental illness in the City of St. Louis, using de-identified ED discharge records obtained from the Missouri Department of Health and Senior Services (DHSS). The study included all EDs across healthcare facilities in the City of St. Louis, from 1 January 2018 to 31 December 2021. Each emergency department visit within the city limits was linked to a census tract using residential addresses on hospital intake and discharge data. The initial data cleaning process removed any cases with missing information, addresses, or cases outside of city limits. This exclusion ensures more reliable and accurate spatial accuracy for analysis. Only visits occurring at emergency departments located within the City of St. Louis were included, and visits by residents located outside of the city were excluded. This approach focuses on service utilization within the city’s healthcare infrastructure.

We fit three models that account for the spatial and temporal autocorrelation that is present in the data. These models helped us evaluate whether temporal trends were best captured by year-specific effects, an overall pandemic effect, or temporal smoothing via a random walk. This approach allowed us to fit a hierarchical Poisson model under reasonable assumptions about the random error.

To assess the potential impact of the COVID-19 pandemic, the study period was divided into two timeframes: pre-COVID-19 (1 January 2018 to 31 December 2019) and during COVID-19 (1 January 2020 to 31 December 2021). The discharge data included information on ED visits, ICD-10-based medical diagnoses, and relevant demographic characteristics of the patients. The ICD codes for the various categories of mental health are shown in Table 1. A patient who had multiple ICD diagnoses was counted in each category, although most patients had one or at most two mental health diagnoses.

### 2.2. Study Sample

The study sample included all emergency department (ED) visits during the study period by women aged 15 to 49 with a diagnosis of mental illness who resided in the City of St. Louis. Mental illness was identified using ICD-10 diagnosis codes and categorized into eight clinically coherent groups: depression, bipolar disorder, anxiety disorders, post-traumatic stress disorder, obsessive–compulsive disorder, psychotic disorders, substance use disorders, and other mental health conditions. Diagnostic groupings were based on shared symptom profiles and established conventions in emergency department mental health surveillance, consistent with the Centers for Disease Control and Prevention’s National Syndromic Surveillance Program (NSSP), which aggregates ICD-10 mental health diagnoses into clinically meaningful categories while retaining residual groupings for infrequent or heterogeneous conditions. Less prevalent diagnoses, including post-traumatic stress disorder and obsessive–compulsive disorder, were retained as distinct categories to preserve clinical interpretability while maintaining sufficient sample sizes for reliable statistical analysis. The residual “other” category captures diagnoses that did not fit primary groupings, a common practice in ED-based mental health research.

### 2.3. Measures

The primary outcome of this study was the number of emergency department visits by residents of each census tract. Given that the unit of analysis is the census tract, this study employs an ecological design [37,38]. All covariates, measured at the census tract level, were derived from the U.S. Census Bureau’s American Community Survey (ACS) 5-year estimates, which provided temporally consistent measures across the study period. The covariates used were percentage of residents with private insurance, percentage with public insurance, median age, median household income, and percentage of adults with at least a high school diploma. The percentages reported are the raw unscaled percentages on a scale of 0% to 100%. These data were linked to each ED visit to characterize neighborhood socioeconomic conditions associated with emergency department utilization for mental health among women of reproductive age. The ED hospital discharge database is visit-based rather than patient-based, meaning individuals could be included multiple times if they had more than one ED visit during the study period. The study did not distinguish between first-time and repeat visits.

## 3. Analytical Approach

The analytical units for this study are the census tracts located within the boundaries of the City of St. Louis. (Notably, the City of St. Louis is independent of St. Louis County and any other county in Missouri; it functions as its own county for most administrative purposes.)

The census tracts are arbitrarily drawn regions defined by the Census Bureau. Differing boundaries could, of course, lead to different results. This is called the modifiable area unit problem (MAUP) [38]. The regions defined by the census tracts are generally small and are therefore somewhat homogeneous in many of the relevant social variables. Most census tracts lie completely within established neighborhoods of the city.

Because the units of analysis are geospatial areas rather than individual patients, the study employs an ecological framework. Caution is required when interpreting the findings, as ecological studies do not account for individual-level variation. For example, one might observe that areas with lower median income tend to have higher volumes of mental health-related emergency department visits; however, this does not imply that all individuals with lower income are more likely to use the ED for mental health concerns; we cannot say that people with low incomes tend to have a higher frequency of mental health-related emergency department visits.

Moran’s *I*, described in the next section, indicates the presence of spatial autocorrelation. Because the time dimension is also present, we fit a number of spatio-temporal models for the occurrence of ED visits by census tract. The following notation is helpful for describing the various models. Let $Y_{it}$ denote the number of ED visits for mental illness (or some particular subcategory of mental illness) in census tract *i* at time *t*. We assume that

$$Y_{it}\;∼\;\mathrm{POISSON}\left(P_{i}\lambda_{it}\right)$$

where $\log\lambda_{it}$ can take on various forms, depending on the assumed structure of the spatio-temporal model, and $P_{i}$ is the population at risk in census tract *i*. This population included women aged 15 to 49 who resided in census tract *i* in the City of St. Louis during the study period. Population size estimates were based on 2020 data, which were assumed to remain constant throughout the duration of the study. Each statistical model includes two random effect error terms to account for spatial autocorrelation and independence—referred to as correlated heterogeneity and uncorrelated heterogeneity, respectively. The models differ in the assumptions they make regarding the effect of time on the outcome.

The predictor variables are measures of social conditions across the census tracts, which we might expect to be correlated. To address the level of multicollinearity, we computed the variance inflation factor (VIF) for each predictor. Values of VIF between 5 and 10 indicate moderate multicollinearity and values of 10 or higher indicate strong multicollinearity. Most of the VIFs shown in Table 2 are between 5 and 6.5, which suggests a moderate degree of multicollinearity.

### 3.1. Model 1: Indicator Variables for the Years

For yr =18,19,20,21 (representing years 2018, 2019, 2020, and 2021, respectively), define

$$x_{1,\mathrm{yr}}=\left\{\begin{array}{cc} 1, & \mathrm{if}\mathrm{year}=\mathrm{yr}\\ 0, & \mathrm{otherwise}. \end{array}\right.$$

The log of the rate $\lambda_{it}$ is then

(1) $$\log\lambda_{it}=\beta_{0}+x^{\prime }\beta +\alpha_{19}x_{1,19}+\alpha_{20}x_{1,20}+\alpha_{21}x_{1,21}+s_{i}+v_{i}$$

where $x^{\prime }$ is a vector of census-tract-level covariates and β is the corresponding vector of regression coefficients. The parameters $\alpha_{19},\;\alpha_{20}$, and $\alpha_{21}$ are the coefficients for the year indicator variables. The uncorrelated random effects satisfy

$$v_{1},v_{2},\ldots ,v_{n}\;∼\;i.i.d.N\left(0,\sigma_{v}^{2}\right)$$

where *n* is the number of census tracts in the City of St. Louis. The spatially autocorrelated random effects $s_{1},s_{2},\ldots ,s_{n}$ have zero mean but with a covariance matrix that depends on the neighborhood structure of the census tracts. The conditional distribution of $s_{i}$, given the values $s_{j}$ for region *i*’s neighbors (indexed by *j*), is

$$s_{i}\;|\;\left(s_{j}\in \begin{array}{c} \mathrm{neighbors}\;\mathrm{of}\;\mathrm{region}\;i \end{array},\;\sigma_{s}^{2}\right)\;∼\;N\left({\bar{s}}_{i},\frac{\sigma_{s}^{2}}{m_{i}}\right)$$

where $m_{i}$ is the number of regions adjacent to region *i* and

$${\bar{s}}_{i}=\frac{1}{m_{i}}\sum_{j\;\in \;\mathrm{nghbrs}\;\mathrm{of}\;i}\;s_{j}.$$

This conditional specification determines the unconditional distribution of the vector s, which is a multivariate normal distribution whose covariance matrix has rank n−1 because the $s_{i}$ are constrained to sum to 0. See Refs. [38,39,40] for a further discussion about how the neighborhood structure determines the covariance matrix for the spatial random effects $s_{i}$. In effect, the correlation structure of the vector s allows for a (usually positive) correlation between the outcomes of neighboring regions. Let $\tau_{v}=1/\sigma_{v}^{2}$ and $\tau_{s}=1/\sigma_{s}^{2}$ denote the precisions for the i.i.d. random effects $v_{i}$ and the spatially correlated random effects $s_{i}$, respectively.

The model described here is often called the BYM model (after Besag, York, and Mollie [41]) or sometimes the conditional autoregressive (CAR) model. The CAR model for the spatial random effects is based on the neighborhood structure (i.e., the structure that specifies which regions share a boundary with which other regions). An alternative is to assume that the covariance of the spatial random effects for two regions is inversely related to some function of the distance between the two regions. Although the CAR model seems to be more commonly applied in practice, our main reason for selecting it was that the wealthy and poor neighborhoods in St. Louis are often close together and the rich pockets are spread across the city. Because of this, it seems unreasonable that the random effects’ correlation matrix would satisfy the inverse distance metric.

In a hierarchical Bayesian model (like BYM), the parameters can be classified into three groups: fixed effects, random effects, and hyperparameters. The regression coefficients are considered fixed effects, although we do place a prior on them and determine the posteriors. The $v_{i}$ and $s_{i}$ are the random effects because we assume that they are drawn from some prior distribution. The hyperparameters are the parameters of these priors; in this case, the precisions are $\tau_{v}$ and $\tau_{s}$. The parameters for all of the models are shown in Table 3. If we let *k* be the number of predictor variables, i.e., k=dim(β), then the model with indicators for the years has k+4 fixed effects (the intercept, the *k* coefficients, and indicators for the years 2019, 2020, and 2021 using 2018 as a reference), 2n random effects (the $v_{i}$ and $s_{i},\;i=1,2,\ldots ,n$), and two hyperparameters ($\tau_{v}$ and $\tau_{s}$).

To approximate the posterior distributions of the model parameters, we use the integrated nested Laplace approximation (INLA) of Ref. [42]; see also Ref. [39] for a survey of how INLA can be applied to spatial and spatio-temporal models.

We applied the default priors in R’s INLA package [43], which are as follows:
  Intercept $\beta_{0}$N(0,prec=0), i.e., the flat, improper prior  Slope $\beta_{i}$N(0,prec=0.001)  Precision (hyperparameters)gamma(1,0.00005)

We performed a sensitivity analysis for the priors by considering eight combinations of the three priors (for the intercept $\beta_{0}$, the slopes $\beta_{j},\;j\geq 1$, and the precision hyperparameters). The results of these eight regressions are shown in Tables S1–S8 in the Supplementary Materials. The priors seem to have little effect on the estimated parameters.

Since INLA works with the log of the precision, rather than the precision itself, the user must specify the prior for the precision as loggamma(a,b). With the INLA default for a being 1, the default prior is an exponential distribution with mean 1/0.00005=20,000. For the random walk models, the prior for the initial value of the time series is taken to be R’s default, which is a normal distribution with small precision (i.e., large variance).

### 3.2. Model 2: Indicator Variables for COVID-19 Years

Here, we define an indicator variable for the COVID-19 years 2020 and 2021. Specifically,

$$x_{\mathrm{COVID}}=\left\{\begin{array}{cc} 1, & \mathrm{if}\;\mathrm{year}=2020\;\mathrm{or}\;2021\\ 0, & \mathrm{otherwise} \end{array}\right.$$

For Model 2, the log of the rate $\lambda_{it}$ is

(2) $$\log\lambda_{it}=\beta_{0}+x^{\prime }\beta +\alpha_{COVID}x_{COVID}+s_{i}+v_{i}.$$

Here, $v_{i}$ and $s_{i}$ are the uncorrelated and spatially autocorrelated random effects as described in Model 1. The categorization of parameters for Model 2 is shown in Table 3.

### 3.3. Model 3: First-Order Random Walk for Years

Model 3 assumes a random walk term in the model to account for the year-to-year changes in the log of the rate. The model for the number of cases in census tract *i* at time *t* is $Y_{it}\;∼\;\mathrm{POISSON}\left(P_{i}\lambda_{it}\right).$ The log rate satisfies

(3) $$\log\lambda_{it}=\beta_{0}+x^{\prime }\beta +s_{i}+v_{i}+\gamma_{t}+\phi_{t}$$

where the random walk component $\gamma_{t}$ satisfies

$$\gamma_{0}∼p\left(\right),\;\;\gamma_{t}∼N\left(\gamma_{t-1},1/\tau_{\gamma }\right),\;\;t=1,2,3,4$$

and $\phi_{1},\ldots ,\phi_{4}∼i.i.d.\;N\left(0,1/\tau_{\phi }\right)$. The value of $\gamma_{t}$ can be thought of as being the previous year’s random effect $\gamma_{t-1}$ plus an independent random effect centered at 0. The parameters of Model 3 are shown in Table 3.

Models 1 through 3 were fit for the purpose of identifying factors that are associated with ED visits by women of reproductive age. The approach we took does not give detailed information about the change in the rate of ED visits across time. For this purpose, we fit Models 4 and 5 across months, rather than years. The model that uses monthly data and a first-order random walk together with a space–time interaction can be used for surveillance, since it provides a way to predict the number of cases for the next month. Observed values outside the prediction would provide evidence of an outbreak of mental illness.

### 3.4. Model 4: First-Order Random Walk for Months

Although the models for years provide a good basis for studying the effect of predictor variables and the effect due to COVID-19, they provide little detail about the short-term change in the mental health-related ED visit outcome over time. If we would like to provide finer details about the change in the outcome over time, we should make the time interval shorter than one year. We aggregated by month from the beginning of 2018 to the end of 2021 for this purpose. With T=48 time periods, the random walk model is more appropriate than a model with a fixed effect for each month (which would involve 47 fixed parameters, using January 2018 as the reference).

The model for months is then $Y_{it}\;∼\;\mathrm{POISSON}\left(P_{i}\lambda_{it}\right)$, where

(4) $$\log\lambda_{it}=\beta_{0}+x^{\prime }\beta +s_{i}+v_{i}+\gamma_{t}+\phi_{t}$$

and $\phi_{1},\phi_{2},\ldots ,\phi_{T}$ are i.i.d. $N(0,1/\tau_{\phi })$ random effects. As before, $s_{i}$ and $v_{i}$ are the spatially correlated and uncorrelated random effects, respectively. The random walk component $\gamma_{t}$ satisfies

$$\gamma_{0}∼p\left(\right),\;\;\gamma_{t}∼N\left(\gamma_{t-1},1/\tau_{\gamma }\right),\;\;t=1,2,3,\ldots ,T.$$

This model is the same as Model 3, except that here we have T=48 time periods, thereby providing more detailed information about the short-term change in mental health-related ED visit outcome.

Normally, the model in Equation (4) would produce predicted time series for the various census tracts that do not intersect. This occurs because the effects of space (*i*) and time (*t*) are additive in (4). In our case, however, the predictor variables change slightly at the beginning of each year, so from December of one year to January of the subsequent year, it is possible for there to be crossings in the predicted time series for the *n* census tracts. This happens occasionally.

### 3.5. Model 5: First-Order Random Walk for Months with Space–Time Interaction

Model 4 assumes that all census tracts move together across time (except at year-end when covariates change), precluding, for example, the possibility that the change from one month to the next may be positive for some regions and negative for others. To overcome this limitation, we introduce a space–time interaction to Model 4.

Blangiardo and Cameletti [44] describe four possible space–time interaction terms that can be added to the model for $\log\lambda_{it}$. The simplest interaction, called Type I, assumes a correlation between the i.i.d. space and time random effects, $v_{i}$ and $\phi_{t}$, respectively. Interactions of Types II, III, and IV involve assuming a correlation between other possible pairs of one space effect and one time effect. We have assumed a Type I interaction by adding an i.i.d. random effect $\delta_{it}$ for all pairs of space *i* and time *t*:

(5) $$\log\lambda_{it}=\beta_{0}+x^{\prime }\beta +s_{i}+v_{i}+\phi_{t}+\gamma_{t}+\delta_{it}$$

where the nT terms $\delta_{it}$ satisfy

$$\underset{\mathrm{census}\;\mathrm{tract}\;1}{\underset{︸}{\delta_{1,1},\delta_{1,2},\ldots ,\delta_{1,T}}}\;,\;\ldots \;,\;\underset{\mathrm{census}\;\mathrm{tract}\;n}{\underset{︸}{\delta_{n,1},\delta_{n,2},\ldots ,\delta_{n,T}}}\;∼i.i.d.\;N\left(0,1/\tau_{\delta }\right).$$

## 4. Results

**Figure 2:**
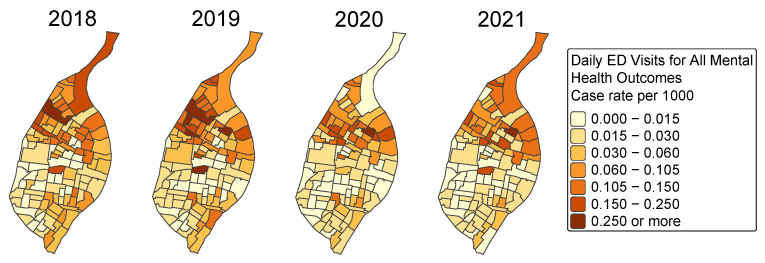
Choropleth maps for the daily case rate for all mental health outcomes, 2018–2021.

**Figure 3:**
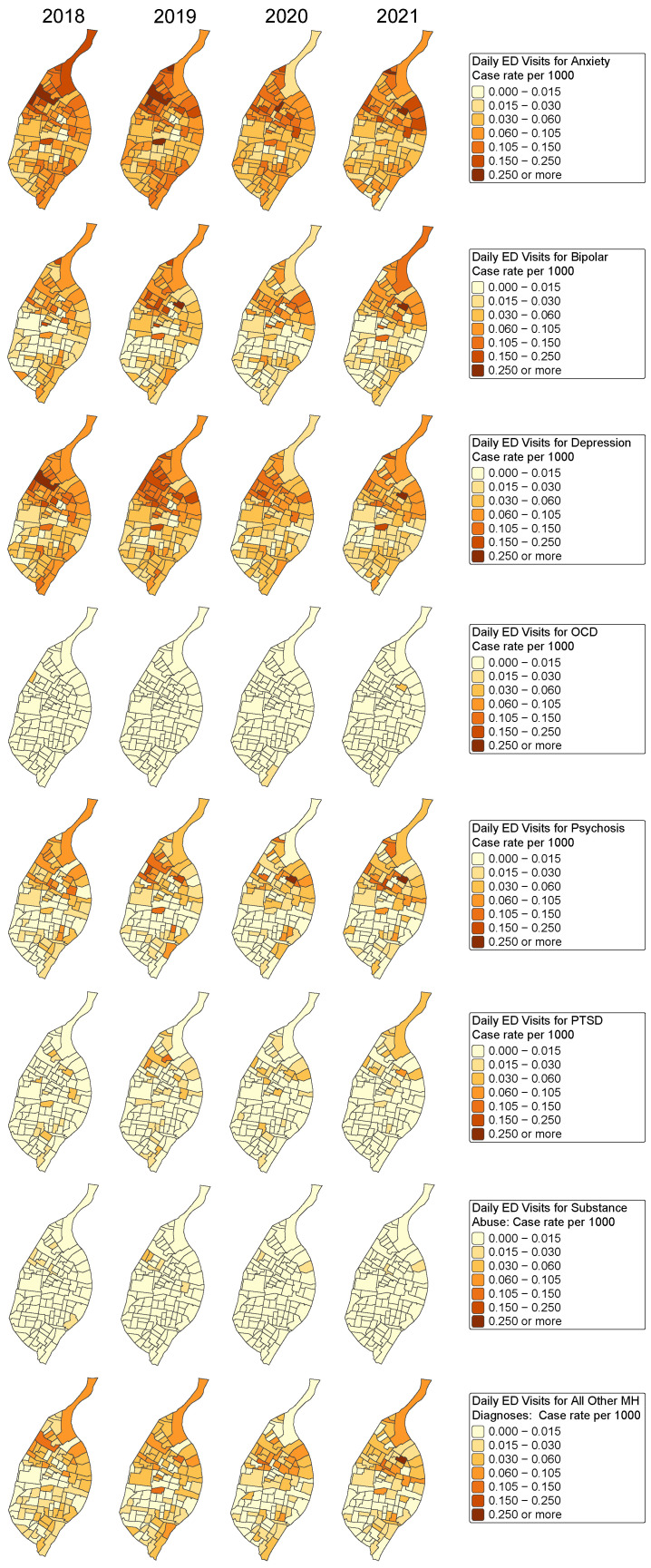
Choropleth maps for outcome variables in the spatio-temporal models, 2018–2021.

Our outcome is the number of ED visits by census tract due to mental illness among women aged 15 to 49 years of age from the city of St. Louis in the timeframe 2018–2021. Mental illness cases were categorized into eight groups as shown in Table 4 for the pre-COVID-19 (2018–2019) and during-COVID-19 (2020–2021) study periods. Figure 2 shows choropleth maps for the mental illness-related ED visits for all categories of mental health, across the census tracts within the city of St. Louis for the years 2018–2021. We can see a clear drop in the daily case volume from 2019 to 2020, indicating less acute care usage during the early COVID-19 pandemic (Figure 2). Choropleth maps for the various causes are shown in Figure 3.

To assess whether the census tract data are autocorrelated, we computed Moran’s I statistic for each category of mental illness as well as all cases aggregated. Moran’s I is used to assess the spatial autocorrelation in a data set involving rates in various regions. If the rates in neighboring regions are similar, there is positive autocorrelation and Moran’s I will tend to be positive. This is common in models for disease counts. If the rates in neighboring regions are negatively correlated, then Moran’s I will tend to be negative; this situation is rare, especially in epidemiology. The formula for Moran’s I [45] looks similar to the formula for the sample correlation coefficient, although it differs somewhat. Under the assumption of independence across the regions, the expected value of Moran’s I is −1/(N−1), where *N* is the number of regions. The minimum and maximum possible values of I are dependent on the neighborhood structure, but the minimum and maximum are near −1 and 1, respectively. The results for Moran’s I, shown in Table 5, provide evidence of spatial autocorrelation for all categories except OCD, which had very small sample sizes.

The predictor variables used for the five models described in the Methods Section include the following census-tract-level variables:

% private insurance
% public insurance
median age
median income
education, defined as % with a high school diploma.

**Figure 4:**
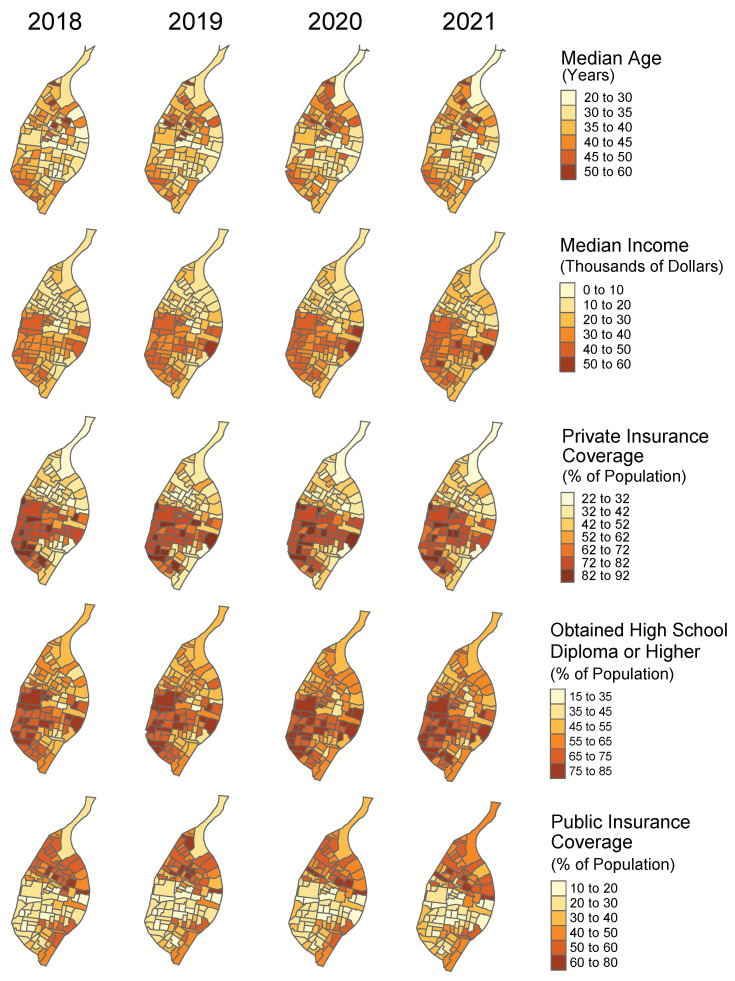
Choropleth maps for predictor variables in the spatio-temporal models, 2018–2021.

Choropleth maps for these covariates across years are shown in Figure 4. These covariates change yearly, although the year-to-year change is usually small.

We fit Models 1, 2, and 3 to each mental illness category as well as to the aggregated total. Table 6 shows the coefficient estimates for the census-tract-level variables described above when the response is all mental health outcomes, while Table 7 does the same for anxiety, depression, and substance abuse. Choropleth maps for these outcomes are shown in Figure 3. Tables of the Poisson regression estimates for other mental health outcomes are shown in Tables S9–S13 in the Supplementary Materials. Some of the categories, for example OCD, PTSD, and substance abuse, had small counts, so the estimated regression parameters have less precision and therefore wider credible intervals. It is thus more difficult to assess the effect of the predictor variables on ED visits for OCD because of the small sample sizes.

For the aggregated outcome of emergency department visits related to all mental health diagnoses, all three models showed negative coefficients for the percentage of residents with private insurance. The credible intervals for these estimates included only negative values, indicating that census tracts with a higher proportion of privately insured residents experienced lower rates of mental health-related acute care utilization. In contrast, the coefficients for the percentage of residents with public insurance were positive; however, their credible intervals included zero, suggesting insufficient evidence to conclude an association between public insurance coverage and mental health-related ED utilization at the census tract level. The % Private Insurance and % Public Insurance do not add up to 100% because there is another category, “uninsured,” that is a sizable percentage for many census tracts.

Additionally, coefficients for census tract median age and education level were positive, with credible intervals that excluded zero. This suggests that census tracts with older populations or higher levels of educational attainment tend to exhibit higher volumes of mental health-related ED visits. In contrast, income coefficients were consistently negative across models, with credible intervals excluding zero, indicating that census tracts with higher median incomes are associated with lower utilization rates of acute care for mental illness.

Table 7 also shows that the coefficients and credible intervals for the substance abuse category show patterns similar to those observed for the aggregated mental health-related outcome. In all three models, the percentage of residents with private insurance has a negative association with substance abuse-related ED visits, and the credible intervals exclude zero, indicating a statistically significant effect. For median age, median income, and education, the signs of the estimated coefficients are consistent with those for the aggregated outcome; however, the credible intervals for these variables include zero, suggesting a lack of statistical significance. Substance abuse was the second-least common mental illness category in the study, with 150 recorded cases before the COVID-19 pandemic and 97 cases during the pandemic period.

The above analysis is based on the assumption of a Poisson model for counts. The Poisson distribution has just one parameter, which is both the mean and the variance. When the variance exceeds the mean, we have the phenomenon called overdispersion. If overdispersion is suspected, the negative binomial distribution is often assumed as the model for the outcome. We ran the models using INLA with the negative binomial distribution for the outcome. The results of the regression for all mental health outcomes are shown in Table 8. Tables S14–S22 in the Supplementary Materials show the results of negative binomial regression applied to the various categories of mental health. While some of the coefficients have changed in magnitude from the Poisson to the negative binomial model, the general conclusions remain the same. The Supplementary Materials show the results for the other mental health outcomes.

In hierarchical Bayesian models such as those used in this study, model fit is commonly evaluated using the deviance information criterion (DIC) [46]. The DIC penalizes models with a greater number of effective parameters and is scaled such that lower values indicate a better model fit. Models whose DICs differ by 2 or less are considered to have a similar degree of fit. The DIC for Models 1 through 3 are shown in Table 9. For the aggregated mental health outcomes, the DIC values for Model 1 (year indicator) and Model 3 (random walk) are nearly identical, while Model 2 (COVID-19 indicator) yields a substantially higher DIC. This suggests that, although the COVID-19 pandemic may have had an effect, the data support a more nuanced temporal interpretation than a simple pre-/post-COVID-19 indicator. Across all mental health categories—except for obsessive–compulsive disorder (OCD), which had a very small sample size—Model 1 (year indicator) consistently produced the lowest DIC, indicating the best model fit, although some DICs are within two units, suggesting a similar fit.

To gain some understanding of the practical effects of the predictor variables, we considered the effect of a single predictor variable moving from its first quartile to its third quartile. The expected value of the number of cases $Y_{it}$ in census tract *i* at time *t* is

$$E\left(Y_{it}\right)=P_{it}\exp\left(\beta_{0}+\beta_{1}x_{it1}+\beta_{2}x_{it2}+\cdots +\beta_{p}x_{itp}\right)$$

where $x_{itk}$ is the value of predictor variable *k* at time *t* for census tract *i*. If we fix all predictor variables except variable *k*, then the ratio of the expected value at levels $x_{k,\mathrm{low}}$ and $x_{k,\mathrm{high}}$ is

$$\frac{P_{it}\exp\left(\beta_{0}+\beta_{1}x_{it1}+\beta_{2}x_{it2}+\cdots +\beta_{k}x_{k,\mathrm{high}}+\cdots +\beta_{p}x_{itp}\right)}{P_{it}\exp\left(\beta_{0}+\beta_{1}x_{it1}+\beta_{2}x_{it2}+\cdots +\beta_{k}x_{k,\mathrm{low}}+\cdots +\beta_{p}x_{itp}\right)}=e^{\beta_{k}\left(x_{k,\mathrm{high}}-x_{k,\mathrm{low}}\right)}.$$

If we let $x_{k,\mathrm{high}}$ and $x_{k,\mathrm{low}}$ denote the third and first quartiles of the predictor variable $x_{k}$, then we can estimate the effect of moving from the first to the third quartile as

$$\mathrm{estimate}\;\mathrm{of}\;\mathrm{effect}=e^{{\hat{\beta }}_{k}\left(x_{k,Q_{3}}-x_{k,Q_{1}}\right)}.$$

This is the multiplicative effect of variable *k*, so that a value of 1.5 indicates an increase of 50% as variable $x_{k}$ moves from $Q_{1}$ to $Q_{3}$.

Table 10 shows these values for Model 1 applied to all mental health outcomes. Other models for other diagnoses are similar. By this measure, the percentage of public insurance had the greatest effect, as it increased by nearly 36% the predicted number of cases of ED visits as the percentage of those with public insurance went from the 25 percentile (24.1) to the 75th percentile (50.3).

To explore short-term variability in mental health-related ED visits, we fit models to monthly aggregated data. Model 4 was identical to Model 3, except that it utilized monthly rather than yearly data. Model 5 extended this by incorporating a space–time interaction term. For the “all mental health-related outcomes” category, we present time series plots comparing the raw data, the monthly spatio-temporal model (Model 4), and the spatio-temporal model with space–time interaction (Model 5).

When covariates are held constant, the time series plots from the model without space–time interaction are expected to move in parallel, without the curves crossing. However, since covariate values changed annually, some crossings occur at the transition between calendar years. The addition of the space–time interaction in Model 5 allows for greater flexibility, enabling changes in mental health-related ED utilization to vary across census tracts over time.

**Figure 5:**
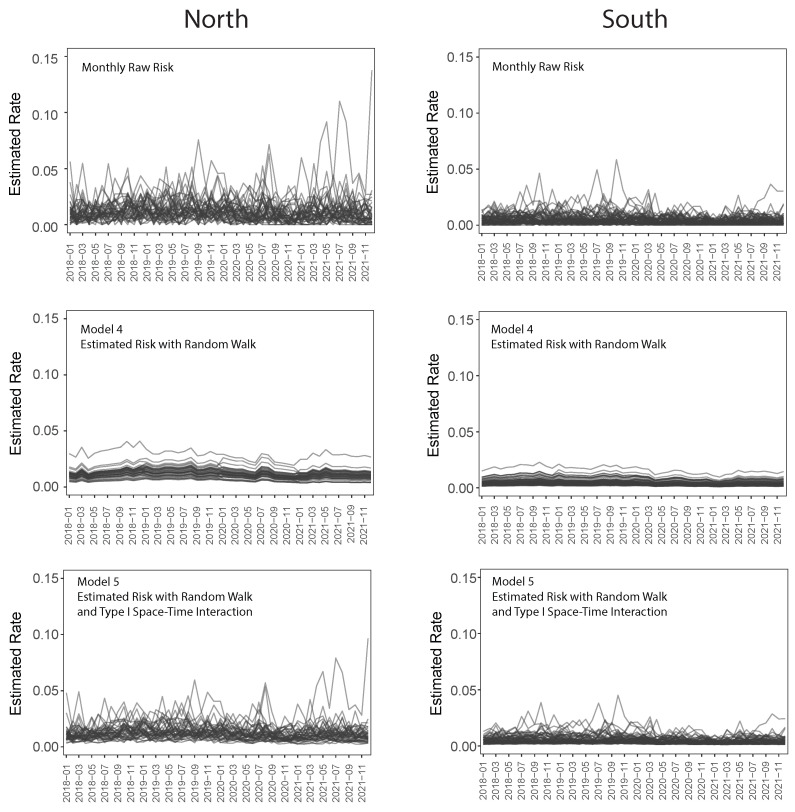
Rates of hospitalization for “all mental health outcomes.” Raw rates (**top**). Monthly spatio-temporal model (**middle**). Monthly spatio-temporal model with space–time interaction (**bottom**).

The results of Models 4 and 5 are shown in the middle and bottom rows of Figure 5. The left column shows the time series plots for those census tracts that are north of the Delmar divide, while the right column shows those census tracts south of the Delmar divide. Each curve represents a census tract within the City of St. Louis. A noticeable decline in mental health-related ED visits begins near the end of 2019 and continues through April 2020, coinciding with the onset of COVID-19 in the United States and the implementation of social restrictions. Social restrictions remained in effect throughout much of 2020 and the COVID-19 vaccine became available in early 2021. Overall, the number of ED visits for mental health outcomes decreased from 2019 to 2020, and again from 2020 to 2021. In 2021, the census tracts in the north seemed to recover to nearly pre-COVID-19 levels, while those in the south dropped from 2020 to 2021. This phenomenon can be seen in Figure 5 as well as Figure 2.

## 5. Discussion

This study investigated census-tract-level rates of emergency department (ED) utilization for mental health-related concerns among the population of women of reproductive age in the City of St. Louis. We analyzed temporal trends from 2018 to 2021 and examined spatial patterns across tracts to understand how neighborhood characteristics influenced these outcomes before and during the COVID-19 pandemic. Findings indicated a decline in mental health-related ED visits during 2020, the initial year of the pandemic, followed by a rebound in 2021. Geospatial analyses revealed pronounced variation in ED visit rates, with higher utilization concentrated in the northern areas of the City of St. Louis—neighborhoods characterized by lower socioeconomic status and a predominantly African American population. These spatial and temporal patterns underscore enduring area-level structural inequities in mental health service access within census tracts populated by vulnerable urban women of reproductive age. In the context of mental health and substance use, higher emergency department utilization should be understood not as evidence of adequate access to care, but rather as a marker of crisis-driven, last-resort service use that reflects unmet need and limited access to timely, longitudinal outpatient treatment. Understanding these tract-level disparities is critical for informing place-based behavioral health interventions and guiding equitable resource distribution to high-need geographic areas, particularly during public health emergencies.

Nationally, ED visits declined during the early phase of the pandemic across most diagnostic categories [47,48], yet visits related to mental health and substance use disorders declined less sharply and, in some cases, increased as a proportion of total visits. Similarly, telehealth visits increased substantially for behavioral health crises during COVID-19 [49,50]. ED encounters for substance use, especially those involving opioids, often remained near or above pre-pandemic levels [51,52,53,54]. Common reasons for substance-related visits included opioid overdoses, alcohol-related harm, and cigarette use, with opioid-related presentations notably exceeding prior baselines. These national trends underscore the persistent unmet behavioral health needs in communities and highlight the ways in which crises such as COVID-19 can amplify structural vulnerabilities. However, few studies have examined how these patterns played out within cities geographically, across different neighborhoods, or among women in the broader reproductive-age range. Among women of reproductive age, neighborhood disadvantage may intersect with reproductive and perinatal care pathways, including insurance churn, disruptions in prenatal and postpartum coverage, and limited access to outpatient mental health services during early adulthood. These dynamics may help explain why spatially concentrated patterns of mental health-related ED utilization persist among women in their 20 s and 30 s, even as overall ED use declined nationally during the early pandemic. Importantly, these national findings are cited for contextual comparison; the present analysis focuses on mental health-related ED visits within St. Louis and does not include total all-cause ED visit counts, precluding direct estimation of proportional changes relative to overall ED utilization.

To capture these dynamics in a spatially and temporally nuanced way, we implemented a series of spatio-temporal models that varied in complexity and underlying assumptions. These included fixed year effects, a binary COVID-period indicator, first-order random walks (yearly and monthly), and models incorporating space–time interaction terms. Each model offered distinct advantages in detecting temporal trends, spatial heterogeneity, or the interaction between the two. Performance was assessed using the deviance information criterion (DIC), which helped balance model fit with parsimony. While all models contributed meaningful insights, the more granular monthly models, particularly those incorporating space–time interactions, were most effective at detecting short-term fluctuations and localized disparities. This comparative modeling analysis not only enhanced our understanding of spatial variations in ED use among women of reproductive age during the pandemic but also demonstrated the utility of flexible, multi-scale modeling frameworks in area-level public health surveillance.

Findings from this analysis revealed both anticipated and revealing patterns. A sharp decline in ED visits occurred during the early months of the pandemic, beginning in early 2020 and continuing through mid-year. This decline was consistent across multiple modeling approaches and aligned with national reports of deferred or avoided care during the height of pandemic-related restrictions. However, spatial variation in utilization persisted throughout the study period. Some census tracts exhibited relatively stable or even rebounding rates of ED visits in later months, suggesting that localized vulnerabilities and barriers to outpatient care shaped patterns of service use independent of broader temporal trends.

Importantly, tract-level socioeconomic characteristics were strongly associated with rates of ED utilization for mental health reasons among the population of women of reproductive age. Census tracts with higher rates of private insurance coverage and greater median household income consistently exhibited lower rates of emergency care utilization for mental illness among this demographic, suggesting that these geographic areas may have had better access to outpatient care or greater community-level resources to reduce reliance on EDs. Conversely, census tracts with higher rates of public insurance, younger age distributions, and lower educational attainment experienced elevated ED utilization rates among women of reproductive age, highlighting structural inequities in behavioral health access at the neighborhood level. These likely reflect structural barriers to outpatient mental healthcare such as limited provider availability, reimbursement constraints, and care fragmentation. These patterns are consistent with prior national studies showing higher emergency department utilization among Medicaid and Medicare beneficiaries compared with commercially insured adults, particularly for conditions that could be managed in outpatient care, reflecting persistent access and system-level barriers rather than insurance effects alone [55,56,57]. These associations held even during periods of overall reduced utilization, reinforcing the idea that social determinants of health shape not only the geographic distribution of healthcare demand, but also the spatial patterns of where and when care is accessed. The spatial patterns reflected in Figure 2 and Figure 3 are consistent with well-documented geographic disparities within St. Louis, most notably the divide between neighborhoods north and south of Delmar Boulevard, often referred to as the “Delmar Divide.” For example, persistently elevated depression-related ED utilization in specific neighborhoods may indicate a need for expanded outpatient mental health capacity, community-based mental health worker or peer support programs, and improved access to telehealth services for individuals with mild to moderate symptoms. In addition, the observed associations between insurance composition and ED utilization highlight policy opportunities to strengthen behavioral health access for publicly insured and uninsured populations, including partnerships among federally qualified health centers, local health departments, and healthcare delivery systems to improve care coordination and reduce reliance on emergency departments for crisis-driven mental healthcare.

The application of spatial analytic tools provides actionable insights for health system planning and public health intervention at the neighborhood level. Hospital systems can use the identified tract-specific temporal and spatial trends to guide surge staffing, resource allocation, and crisis response strategies tailored to areas with high volumes of ED visits among women of reproductive age. Similarly, behavioral health providers may leverage mapped utilization rates to inform targeted outreach, satellite clinic placement, and community partnership development within underserved census tracts. Although the study period includes the COVID-19 pandemic, the spatial inequities uncovered suggest enduring structural issues that remain relevant beyond periods of crisis.

Public health departments and cross-sector collaboratives can integrate spatio-temporal modeling into broader strategies for surveillance, planning, and health equity. These tools can guide where mobile crisis units are deployed, where behavioral health navigators are placed, or which neighborhoods should be prioritized for service integration. Community advisory boards, behavioral health coalitions, and maternal health collaboratives can use the modeling outputs to advocate for resources, align stakeholder efforts, and ensure interventions are geographically matched to need. Importantly, while this study focused on behavioral health, the geospatial framework developed here can be applied to other outcomes, such as chronic disease management, maternal and infant health, or preventive service delivery. The use of routinely collected administrative data, analyzed through spatial and temporal lenses, offers a scalable and transferable approach for identifying disparities and supporting more equitable, data-driven public health planning in cities across the country.

## 6. Strengths and Limitations

This study has several notable strengths. It leverages objective hospital discharge data encompassing all mental health-related ED visits among women of reproductive age in St. Louis City during a four-year period spanning the COVID-19 pandemic, minimizing selection and recall bias inherent in self-reported data. The application of advanced geospatial methods, including multiple spatio-temporal modeling approaches, revealed significant census-track-level geographic disparities in mental health-related healthcare utilization that would remain obscured in traditional city-level aggregate analyses. The comparative modeling framework, assessed using deviance information criteria, allowed for rigorous evaluation of different temporal structures and demonstrated the utility of flexible analytical approaches for census-track-level public health surveillance. The integration of census-tract-level sociodemographic data through the Census Bureau American Community Survey enabled examination of neighborhood-level structural factors influencing ED utilization patterns by census tract, while the monthly granularity of Models 4 and 5 (Figure 5) captured short-term fluctuations in ED utilization by census tract that annual models would miss. The methodological framework demonstrated here is adaptable beyond the pandemic context and transferable to other health outcomes, offering a scalable model for surveillance and equity-focused public health planning by census tract in urban settings nationwide.

However, several limitations warrant consideration. Most importantly, the ecological study design with census tracts serving as the units of analysis, precludes causal inference, and introduces the possibility of ecological fallacy. It is noteworthy that while neighborhood data can help pinpoint which areas need the most help, this neighborhood-based approach does not show exactly which individuals within those neighborhoods are at the highest risk. Area-based intervention strategies can sometimes lead to inefficient resource allocation by providing services to residents who are not at high risk. Our findings may be limited by potential selection bias as the data from the present study represent ED utilization within the city’s healthcare system and may not capture the complete picture of care-seeking behavior for residents who travel outside city limits for emergency mental health services, which could differentially affect estimates across census tracts if cross-boundary care-seeking varies by neighborhood characteristics. The reliance on ICD-10 diagnosis codes from administrative discharge data introduces the possibility of diagnostic misclassification, as coding practices may vary across providers and facilities, mental health diagnoses may be underreported when patients present with multiple comorbidities, and the primary reason for visit may not fully capture the complexity of behavioral health needs. Unmeasured confounding is another potential limitation of the present study. Other factors influencing ED utilization patterns by census tract, including direct measures of race/ethnicity at the tract level, neighborhood-level availability of outpatient mental health services, proximity to EDs, transportation access, childcare availability, and social support networks, are not captured in the available covariates. Data from the present study also cannot distinguish between first-time and repeat ED visits. As a result, observed patterns of high utilization in certain census tracts may reflect a small number of reproductive-age women with frequent encounters rather than widespread population-level need. Additionally, population denominators were based on 2020 census data and held constant throughout the study period, which may not accurately reflect actual population shifts during 2018–2021, particularly migration patterns potentially accelerated by the pandemic.

It is possible that the hospital discharge data may capture only ED visits resulting in documented discharge and do not include individuals who left without being seen (LWBS) or who self-discharged before receiving care. If LWBS rates vary systematically across census tracts, due to differences in wait times, competing obligations such as childcare, employment, or other reasons, our estimates may underrepresent the true burden of unmet mental health needs in certain neighborhoods, potentially underestimating disparities in areas where barriers to completing ED visits are greatest. In addition, the study period is limited to four years (2018–2021), with only two years of pre-pandemic baseline data. This relatively short timeframe constrains our ability to assess the stability of spatial patterns over longer periods or to distinguish pandemic-related changes from secular trends in mental health service utilization. As a result, the generalizability of our findings to other time periods remains uncertain. Furthermore, while the American Community Survey provides valuable census-tract-level measures, it does not capture individual-level characteristics such as employment status, family structure, social support networks, medication adherence, or personal experiences with discrimination and trauma. This reliance on area-level measures means we cannot account for within-tract heterogeneity in individual risk factors. Our findings may be further limited by residual confounding due to unmeasured confounding by individual-level characteristics that influence both mental health status and ED utilization patterns.

The geographic scope of our study is limited to St. Louis City, which could potentially limit the generalizability of our study findings. Nonetheless, the spatial patterns of inequality by census tract observed likely mirror dynamics in other segregated urban centers where historical policies continue to shape contemporary health disparities. Finally, the study focuses exclusively on ED utilization and does not capture mental health needs addressed through outpatient care, telehealth, or left entirely unmet. Hence, the observed decline in ED visits during early 2020 may reflect genuine reductions in acute crises, deferral of care, or shifts to alternative service modalities. Despite these limitations, the identification of persistent geographic disparities by census tract, particularly the concentration of high ED utilization in predominantly Black, economically disadvantaged neighborhoods, highlights structural inequities at the census tract level, requiring policy attention and providing actionable insights for health system planning and resource allocation.

## 7. Conclusions

This study illustrates the utility of integrating geospatial data with advanced modeling techniques to inform more equitable and responsive mental health system planning, particularly during public health emergencies such as the COVID-19 pandemic. The findings underscore persistent area-level structural disparities in mental healthcare access across census tracts populated by women of reproductive age in urban settings. The data on ED visits for mental health outcomes exhibits autocorrelation across space (since neighboring census tracts tend to have similar rates) and time (since the rate in one period is highly correlated with the rate in the previous period). The models we fit account for both spatial and temporal autocorrelation. By capturing both temporal trends and neighborhood-level variations in emergency department utilization, the analytical approach provides actionable insights for data-driven strategies that target high-need populations and geographies. Despite the models being complex, we believe the spatial and temporal autocorrelations must be accounted for in any analysis.

We applied the spatio-temporal models over yearly and monthly time periods. The yearly time periods gave us information about the effects of the predictor variables on the rate of ED visits for various mental health diagnostic codes. The monthly data gave us finer information about the short-term change in rates; this gave us information about the COVID-19 effect. For the yearly data, the model with a year indicator and the model with a first-order random walk gave nearly identical values of DIC, a measure of fit that penalizes models with more parameters.

While the study’s ecological design and limited covariate data constrain causal interpretation, the spatial patterns identified remain valuable for informing local place-based interventions. Importantly, the modeling framework demonstrated here is adaptable beyond the pandemic context and can support ongoing behavioral health surveillance, guide area-level resource allocation, evaluate the impact of policy initiatives, and ultimately improve the resilience and equity of mental health systems within census tracts characterized by high concentrations of vulnerable populations. Identifying priority locations through neighborhood-level analysis tells us where to focus new programs and policies, but we still need individual-level assessments to decide who should receive specific services. To ensure these strategies are effective, future research should combine geographic data with more detailed individual-level information, such as data from electronic health records or surveys, to confirm that neighborhood-based public health interventions are leading to real, measurable improvements for the people who need them most.

## Figures and Tables

**Table 1:** ICD-10 codes for mental health disease categories.

Disease Category	ICD-10 Codes
Depression	F32.0, F32.1, F32.2, F32.3, F32.4, F32.5, F32.8, F32.89, F32.9, F32A,
	F33.0, F33.1, F33.2, F33.3, F33.4, F33.40, F33.41, F33.42, F33.8,
	F33.9, F34.1, F53.0, O90.6, O99.34
Bipolar Disorder	F30.10, F30.11, F30.12, F30.13, F30.2, F30.3, F30.4, F30.8, F30.9,
	F31.0, F31.10, F31.11, F31.1, F31.13, F31.2, F31.30, F31.31, F31.32,
	F31.4, F31.5, F31.60, F31.61, F31.62, F31.63, F31.64, F31.70, F31.71,
	F31.72, F31.73, F31.74, F31.75, F31.76, F31.78, F31.81, F31.89, F31.9,
	F34.0, F34.81, F34.89, F39
Anxiety	F06.4, F40.9, F40.00, F40.01, F40.10, F40.11, F40.218, F40.240,
	F40.241, F40.8, F41.0, F41.1, F41.3, F41.8, F41.9, F43.0, F45.8, F48.8,
	F48.9, F93.8, F99, R45.7
PTSD	F43.10, F43.11, F43.12
OCD	F42.2, F42.3, F42.4, F42.8, F42.9, R46.81
Psychosis	F06.0, F06.2, F20.0, F20.1, F20.2, F20.3, F20.5, F20.81, F20.89, F20.9,
	F21, F22, F23, F24, F25.0, F25.1, F25.8, F25.9, F28, F29, F53.1, F44.0,
	F44.1, F44.2, F44.81, F44.89, F44.9, F48.1, F48.2
Substance Abuse	F10, F11, F12, F13, F14, F15, F16, F17, F18, F19, O99.320, O99.321,
	O99.322, O99.323, O99.324, O99.325
Other	R45.850, R45.851, F06.1, F06.30, F06.31, F06.32, F06.33, F06.34,
	F34.9, F43.20, F43.21, F43.22, F43.23, F43.24, F43.25, F43.29, F43.81,
	F43.89, F43.9, F44.4, F44.5, F44.6, F44.7, F45.0, F45.1, F45.20, F45.21,
	F45.22, F45.29, F45.41, F45.42, F45.9, F51.01, F51.02, F51.03, F51.04,
	F51.05, F51.09, F51.11, F51.12, F51.13, F51.19, F51.8, F51.9, F54,
	F59, O99.340, O99.341, O99.342, O99.343, O99.344, O99.345

**Table 2:** Variance inflation factors for predictor variables in the hierarchical Poisson regression model.

Predictor Variable	Variance Inflation Factor
Year	1.08
% Private Insurance	6.34
% Public Insurance	6.35
Median Age	2.09
Median Income	5.57
Education (% HS+)	6.00

**Table 3:** Enumeration of the parameters in the various models. Here, k=dim(β), *n* is the number of census tracts, and *T* is the number of time periods.

Model 1:		
Yearly Effect	Parameters	Number of Parameters
Fixed Effects:	$\beta_{0},\;\beta ,\;\alpha_{19},\;\alpha_{20},\;\alpha_{21}$	k+4
Random Effects:	$s_{1},s_{2},\ldots ,s_{n}$; $v_{1},v_{2},\ldots ,v_{n}$	2n
Hyperparameters:	$\tau_{s},\;\tau_{v}$	2
Total		k+2n+6
		
Model 2:		
COVID-19 Year Effect	Parameters	Number of Parameters
Fixed Effects:	$\beta_{0},\;\beta ,\;\alpha_{\mathrm{COVID}}$	k+2
Random Effects:	$s_{1},s_{2},\ldots ,s_{n}$; $v_{1},v_{2},\ldots ,v_{n}$	2n
Hyperparameters:	$\tau_{s},\;\tau_{v}$	2
Total		k+2n+4
		
Model 3:		
Random Walk	Parameters	Number of Parameters
Fixed Effects:	$\gamma_{0},\beta_{0},\;\beta$,	k+2
Random Effects:	$s_{1},\ldots ,s_{n}$; $v_{1},\ldots ,v_{n}$;	
	$\gamma_{1},\ldots ,\gamma_{4}$; $\phi_{1},\ldots ,\phi_{4}$	2n+8
Hyperparameters:	$\tau_{s},\;\tau_{v},\;\tau_{\phi },\;\tau_{\gamma }$	4
Total		k+2n+14
		
Model 4:		
RW for Months	Parameters	Number of Parameters
Fixed Effects:	$\gamma_{0},\beta_{0},\;\beta$,	k+2
Random Effects:	$s_{1},\ldots ,s_{n}$; $v_{1},\ldots ,v_{n}$;	
	$\phi_{1},\ldots ,\phi_{T}$; $\gamma_{1},\ldots ,\gamma_{T}$	2n+2T
Hyperparameters:	$\tau_{s},\;\tau_{v},\;\tau_{\phi },\;\tau_{\gamma }$	4
Total		k+2n+2T+6
		
Model 5:		
RW Months w/Interaction	Parameters	Number of Parameters
Fixed Effects:	$\gamma_{0},\beta_{0},\;\beta$	k+2
Random Effects:	$s_{1},\ldots ,s_{n}$; $v_{1},\ldots ,v_{n}$;	
	$\gamma_{1},\ldots ,\gamma_{T}$; $\phi_{1},\ldots ,\phi_{T}$;	
	$\delta_{1,1},\delta_{1,2},\ldots ,\delta_{1,T},\;\ldots ,$	
	$\delta_{n,1},\delta_{n,2},\ldots ,\delta_{n,T}$	2n+(n+2)T
Hyperparameters:	$\tau_{s},\;\tau_{v},\;\tau_{\phi },\;\tau_{\gamma },\;\tau_{\delta }$	5
Total		k+2n+(n+2)T+7

**Table 4:** Summary of diagnoses before and during the COVID-19 pandemic.

Reason for Visit	Before COVID-19		During COVID-19		Total	
	*n*	(%)	*n*	(%)	*n*	(%)
Anxiety	4120	32.11	3135	32.21	7255	32.15
Bipolar Disorder	1976	15.40	1548	15.90	3524	15.62
Depression	3213	25.04	2085	21.42	5298	23.48
OCD	64	0.50	54	0.56	118	0.52
PTSD	509	3.97	416	4.27	925	4.10
Psychosis	1342	10.46	1149	11.81	2491	11.04
Substance Abuse	146	1.14	97	1.00	243	1.08
Other	1462	11.39	1249	12.83	2711	12.01
Total	12,832	100.00	9733	100.00	22,565	100.00

**Table 5:** Moran’s I, a measure of spatial autocorrelation, for the combined mental health-related ED visit outcomes and for each reason for visit.

Reason for Visit	Time Period	*n*	Moran’s I	*p*-Value
All MH-related	Before COVID-19	12,832	0.295	$3.09\times 10^{-8}$
All MH-related	During COVID-19	9733	0.180	$4.11\times 10^{-4}$
Anxiety	Before COVID-19	4120	0.269	$2.69\times 10^{-7}$
Anxiety	During COVID-19	3135	0.123	0.0100
Bipolar	Before COVID-19	1976	0.152	0.00172
Bipolar	During COVID-19	1548	0.136	0.00482
Depression	Before COVID-19	3213	0.268	$3.59\times 10^{-7}$
Depression	During COVID-19	2085	0.181	$4.38\times 10^{-4}$
OCD	Before COVID-19	64	−0.0522	0.794
OCD	During COVID-19	54	0.108	0.0148
PTSD	Before COVID-19	509	0.261	$8.89\times 10^{-7}$
PTSD	During COVID-19	416	0.0423	0.138
Psychosis	Before COVID-19	1342	0.220	$2.09\times 10^{-5}$
Psychosis	During COVID-19	1149	0.132	0.00524
Substance Abuse	Before COVID-19	146	0.337	$4.88\times 10^{-10}$
Substance Abuse	During COVID-19	97	0.149	0.00238
Other MH-related	Before COVID-19	1462	0.157	0.00164
Other MH-related	During COVID-19	1249	0.130	0.00556

**Table 6:** Ecological spatial models for the three spatio-temporal models applied to all mental health outcomes.

Model	Outcome	Parameter	Estimate	Credible Interval
Model 1: Indicator variable for each year	All MH Outcomes	(Intercept)	−3.96	(−4.46, −3.45)
		Year 2019	0.072	(0.0348, 0.109)
		Year 2020	−0.178	(−0.222, −0.135)
		Year 2021	−0.276	(−0.327, −0.226)
		% Private Insurance	0.000618	(−0.00427, 0.00552)
		% Public Insurance	0.0117	(0.0069, 0.0165)
		Median Age	0.0134	(0.00678, 0.0200)
Model 2: Indicator variable for COVID-19	All MH Outcomes	(Intercept)	−4.08	(−4.57, −3.60)
		COVID-19 Indicator	−0.264	(−0.296, −0.231)
		% Private Insurance	0.00299	(−0.00181, 0.0078)
		% Public Insurance	0.0125	(0.0078, 0.0172)
		Median Age	0.0148	(0.00825, 0.0214)
		Median Income	−0.00529	(−0.0112, 0.000658)
		Education (% HS+)	0.00667	(0.00158, 0.0118)
Model 3: First-order random walk	All MH Outcomes	(Intercept)	−4.03	(−4.55, −3.52)
		% Private Insurance	0.000633	(−0.00427, 0.00555)
		% Public Insurance	0.0116	(0.00685, 0.0164)
		Median Age	0.0133	(0.00674, 0.0200)
		Median Income	−0.0049	(−0.0113, 0.00152)
		Education (% HS+)	0.00722	(0.0021, 0.0124)

**Table 7:** Regression analysis from Poisson model for anxiety, depression, and substance abuse.

Model	Reason for Visit	Predictor Variable	Estimate	(95% Credible Interval)
Model 1: Indicator variable for each year	Anxiety	(Intercept)	−4.180	(−4.900, −3.480)
		Year 2019	0.088	( 0.025, 0.152)
		Year 2020	−0.109	(−0.179, −0.038)
		Year 2021	−0.253	(−0.333, −0.173)
		% Private Insurance	−0.014	(−0.021, −0.007)
		% Public Insurance	0.006	(−0.001, 0.014)
		Median Age	0.010	(−0.000, 0.020)
		Median Income	−0.011	(−0.020, −0.001)
		Education (% HS+)	0.015	( 0.006, 0.023
Model 2: Indicator for COVID-19	Anxiety	(Intercept)	−4.26	(−4.97, −3.56)
		COVID-19 Indicator	−0.219	(−0.273, −0.166)
		% Private Insurance	−0.0115	(−0.0185, −0.00446)
		% Public Insurance	0.00719	(−0.000329, 0.0147)
		Median Age	0.0116	(0.00146, 0.0216)
		Median Income	−0.0121	(−0.0213, −0.00291)
		Education (% HS+)	0.014	(0.00585, 0.0222)
Model 3: First-order random walk	Anxiety	(Intercept)	−4.23	(−4.96, −3.51)
		% Private Insurance	−0.0136	(−0.0206, −0.00647)
		% Public Insurance	0.00637	(−0.00126, 0.014)
		Median Age	0.0101	(−0.0000873, 0.0202)
		Median Income	−0.0000111	(−0.0000207, −0.00000147)
		Education (% HS+)	0.0146	(0.00635, 0.0228)
Model 1: Indicator variable for each year	Depression	(Intercept)	−4.810	(−5.670, −3.980)
		Year 2019	0.049	(−0.023, 0.121)
		Year 2020	−0.281	(−0.365, −0.199)
		Year 2021	−0.530	(−0.627, −0.433)
		% Private Insurance	−0.012	(−0.020, −0.003)
		% Public Insurance	0.009	( 0.001, 0.019)
		Median Age	0.021	( 0.009, 0.033)
		Median Income	−0.000	(−0.011, 0.011)
		Education (% HS+)	0.006	(−0.003, 0.016)
Model 2: Indicator for COVID-19	Depression	(Intercept)	−4.78	(−5.61, −3.97)
		COVID-19 Indicator	−0.41	(−0.473, −0.347)
		% Private Insurance	−0.00943	(−0.0175, −0.00122)
		% Public Insurance	0.00913	(0.000381, 0.0179)
		Median Age	0.0219	(0.01, 0.0338)
		Median Income	−0.00504	(−0.0157, 0.00575)
		Education (% HS+)	0.00615	(−0.00349, 0.0158)
Model 3: First-order random walk	Depression	(Intercept)	−4.81	(−5.67, −3.98)
		Year 2019	0.0491	(−0.0232, 0.121)
		Year 2020	−0.281	(−0.365, −0.199)
		Year 2021	−0.53	(−0.627, −0.433)
		% Private Insurance	−0.0116	(−0.0198, −0.00328)
		% Public Insurance	0.00948	(0.000518, 0.0185)
		Median Age	0.021	(0.0089, 0.033)
		Median Income	−0.0000666	(−0.0114, 0.0114)
		Education (% HS+)	0.00644	(−0.00336, 0.0163)
Model 1: Indicator variable for each year	Substance Abuse (SA)	(Intercept)	−6.420	(−8.440, −4.420)
		Year 2019	0.202	(−0.127, 0.531)
		Year 2020	−0.032	(−0.391, 0.327)
		Year 2021	−0.338	(−0.746, 0.070)
		% Private Insurance	−0.023	(−0.043, −0.002)
		% Public Insurance	0.013	(−0.011, 0.037)
		Median Age	0.012	(−0.022, 0.047)
		Median Income	−0.017	(−0.052, 0.019)
		Education (% HS+)	−0.000	(−0.033, 0.032)
Model 2: Indicator for COVID-19	Substance Abuse (SA)	(Intercept)	−6.42	(−8.44, −4.42)
		COVID-19 Indicator	−0.267	(−0.535, 0.000985)
		% Private Insurance	−0.0211	(−0.0417, −0.000536)
		% Public Insurance	0.0134	(−0.0104, 0.0374)
		Median Age	0.0138	(−0.0201, 0.0482)
		Median Income	−0.018	(−0.0523, 0.0168)
		Education (% HS+)	−0.000674	(−0.0325, 0.0307)
Model 3: First-order random walk	Substance Abuse (SA)	(Intercept)	−6.3	(−8.3, −4.3)
		% Private Insurance	−0.0208	(−0.0413, −0.000234)
		% Public Insurance	0.0114	(−0.0124, 0.0352)
		Median Age	0.0126	(−0.021, 0.0467)
		Median Income	−0.0249	(−0.0578, 0.00823)
		Education (% HS+)	0.0000238	(−0.0312, 0.0308)

**Table 8:** Regression results with a negative binomial model for counts when the outcome is all mental health diagnoses.

Model	Outcome	Parameter	Estimate	Credible Interval
Model 1: Indicator variable for each year	All MH Outcomes	(Intercept)	−3.54	(−4.54, −2.55)
		Year 2019	0.0852	(−0.0225, 0.193)
		Year 2020	−0.168	(−0.282, −0.0532)
		Year 2021	−0.304	(−0.428, −0.179)
		% Private Insurance	−0.0179	(−0.0278, −0.00789)
		% Public Insurance	0.00213	(−0.00903, 0.0133)
		Median Age	0.0234	(0.00889, 0.0381)
		Median Income	−0.00689	(−0.0206, 0.00689)
		Education (% HS+)	0.00326	(−0.00892, 0.0155)
Model 2: Indicator variable for COVID-19	All MH Outcomes	(Intercept)	−3.54	(−4.55, −2.55)
		COVID-19 Indicator	−0.274	(−0.358, −0.188)
		% Private Insurance	−0.0166	(−0.0266, −0.00658)
		% Public Insurance	0.00246	(−0.00874, 0.0136)
		Median Age	0.0242	(0.00959, 0.0389)
		Median Income	−0.00818	(−0.00215, 0.00516)
		Education (% HS+)	0.00279	(−0.00945, 0.015)
Model 3: First-order random walk	All MH Outcomes	(Intercept)	−3.60	(−4.61, −2.60)
		% Private Insurance	−0.0176	(−0.0275, −0.00766)
		% Public Insurance	0.00195	(−0.0092, 0.0131)
		Median Age	0.0231	(0.00862, 0.0377)
		Median Income	−0.00800	(−0.0217, 0.00572)
		Education (% HS+)	0.00323	(−0.00895, 0.0154)

**Table 9:** Deviance information criteria (DIC) for all models on all outcomes. DIC is a measure of overall fit, with smaller values indicating a better fit.

Model	Reason for Visit	Total *n*	DIC
Model 1: Year indicator	All MH-Related Outcomes	22,565	4143.424
Model 2: COVID-19 indicator	All MH-Related Outcomes	22,565	4174.141
Model 3: First-order RW	All MH-Related Outcomes	22,565	4144.117
Model 1: Year indicator	Depression	5298	2479.416
Model 2: COVID-19 indicator	Depression	5298	2509.829
Model 3: First-order RW	Depression	5298	2480.484
Model 1: Year indicator	Bipolar	3524	2495.222
Model 2: COVID-19 indicator	Bipolar	3524	2499.809
Model 3: First-order RW	Bipolar	3524	2496.679
Model 1: Year indicator	Anxiety	7255	2762.544
Model 2: COVID-19 indicator	Anxiety	7255	2780.768
Model 3: First-order RW	Anxiety	7255	2763.203
Model 1: Year indicator	PTSD	925	1584.945
Model 2: COVID-19 indicator	PTSD	925	1604.253
Model 3: First-order RW	PTSD	925	1586.289
Model 1: Year indicator	OCD	118	540.6393
Model 2: COVID-19 indicator	OCD	118	539.0801
Model 3: First-order RW	OCD	118	537.9303
Model 1: Year indicator	Psychosis	2491	2308.833
Model 2: COVID-19 indicator	Psychosis	2491	2339.165
Model 3: First-order RW	Psychosis	2491	2331.068
Model 1: Year indicator	Substance Abuse	243	837.0309
Model 2: COVID-19 indicator	Substance Abuse	243	836.3022
Model 3: First-order RW	Substance Abuse	243	837.5839
Model 1: Year indicator	Other	2711	2295.903
Model 2: COVID-19 indicator	Other	2711	2294.312
Model 3: First-order RW	Other	2711	2297.760

**Table 10:** Estimates of the effect on the number of all mental health outcomes as each predictor moves from its first to third quartile ($Q_{1}$ to $Q_{3}$).

Predictor Variable	Estimate of Slope	Estimate of Effect of Moving from Q1 to Q3
% Private Insurance	0.000618	1.021
% Public Insurance	0.0117	1.359
Median Age	0.0134	1.111
Median Income	−0.00464	0.915
Education (Pct HS+)	0.00732	1.161

## Data Availability

Data for the present study are unavailable due to the sensitive or confidential information related to hospital discharge data used in the analysis. Our code, along with simulated data, is available from the corresponding author.

## Supplementary Materials

The following supporting information can be downloaded at https://www.mdpi.com/article/10.3390/ijerph23020177/s1.

## Abbreviations

The following abbreviations are used in this manuscript:

CAR	Conditional autoregressive model
RW	Random walk
DIC	Deviance information criterion
