# Supplementary material for: Emergency Department Utilization by Women of Reproductive Age for Mental Illness in St. Louis Before and During the COVID-19 Pandemic
Source: Int J Environ Res Public Health. 2026 Jan 30;23(2):177. doi: 10.3390/ijerph23020177 (PMC12941014; doi:10.3390/ijerph23020177)
Supplement: Supplementary file 1 [file ijerph-23-00177-s001.zip › ijerph-3995973-supplementary.pdf]

Supplementary Materials for: Emergency Department Utilization by Women of Reproductive Age for Mental Illness in St. Louis Before and During the COVID-19 Pandemic

Jen Jen Chang, Christopher D. Hopwood, Yuki Sugawara,
Abigail Andresen, Thomas E. Burroughs,
Aya Bou Fakhreddine, and Steven E. Rigdon

The main text of this paper shows the results of the spatio-temporal models fit t o 'all mental health outcomes and one category, 'substance abuse.' The results and conclusions for the other categories of mental health outcomes are nearly the same, except for OCD. The number of ED visits due to OCD was quite small, so the credible intervals are rather wide. For completeness, the full results of the spatio-temporal models are presented in this supplementary materials section.

Section 1 includes Tables S1 – S8 that show the results for the Poisson regression for eight combinations of priors for the intercept β_0, the slopes β_j , $j \geq 1$, and the precision parameters. The selection of the prior has little effect on the estimated coefficients.

Section 2 shows the estimated coefficients of the regression analysis for other mental health outcomes when the outcome is assumed to have a Poisson distribution. This section includes Tables S9 – S13. Before each table, we give the number of ED visits for that diagnosis. The diagnoses with very small counts have wide credible intervals because the sample size is so small.

For the Poisson distribution, the mean and variance are the same. Often, count data exhibit *overdispersion*, which means that the variance exceeds the mean. When this occurs, it is possible to assume a negative binomial distribution for the outcome. Section 3 shows the complete results when the negative binomial is assumed. Discuss whether the NB models yield similar results to the Poisson.

1. Effect of the Prior

Prior for the intercept: $\beta_0 \sim N(0, \text{prec} = 0)$

Prior for the slope parameters: $\beta_i \sim N(0, \text{prec} = 0.001)$, $i = 1, 2, \ldots, p$

Prior for precision parameters: $\tau \sim \text{gamma}(1, 0.00005)$

Model 3:	All MH Outcomes	(Intercept)	-4.03	(-4.55, -3.52)
1st-Order		% Private Insurance	0.000633	(-0.00427, 0.00555)
random walk		% Public Insurance	0.0116	(0.00685, 0.0164)
		Median Age	0.0133	(0.00674, 0.0200)
		Median Income	-0.0049	(-0.0113, 0.00152)
		Education (% HS+)	0.00722	(0.0021, 0.0124)

Table S1: Poisson model with alternate prior(s) applied to all mental health outcomes

Prior for the intercept: $\beta_0 \sim N(0, \text{prec} = 0)$

Prior for the slope parameters: $\beta_i \sim N(0, \text{prec} = 0.001)$, $i = 1, 2, \ldots, p$

Prior for precision parameters: $\tau \sim \text{gamma}(0.1, 0.00001)$

Model 3:	All MH Outcomes	(Intercept)	-4.05	(-4.57, -3.53)
1st-Order		% Private Insurance	0.000716	(-0.00420, 0.00565)
random walk		% Public Insurance	0.0117	(0.00688, 0.0165)
		Median Age	0.0133	(0.00672, 0.0200)
		Median Income	-0.00474	(-0.0112, 0.00169)
		Education (% HS+)	0.00728	(0.00215, 0.0124)

Table S2: Poisson model with alternate prior(s) applied to all mental health outcomes

Prior for the intercept: $\beta_0 \sim N(0, \text{prec} = 0.00001)$

Prior for the slope parameters: $\beta_i \sim N(0, \text{prec} = 0.001)$, $i = 1, 2, \ldots, p$

Prior for precision parameters: $\tau \sim \text{gamma}(1, 0.00005)$

Model 3:	All MH Outcomes	(Intercept)	-4.03	(-4.55, -3.52)
1st-Order		% Private Insurance	0.000629	(-0.00428, 0.00555)
random walk		% Public Insurance	0.0116	(0.00685, 0.0164)
		Median Age	0.0134	(0.00674, 0.0200)
		Median Income	-0.0049	(-0.0113, 0.00152)
		Education (% HS+)	0.00722	(0.00210, 0.0124)

Table S3: Poisson model with alternate prior(s) applied to all mental health outcomes

Prior for the intercept: $\beta_0 \sim N(0, \text{prec} = 0)$

Prior for the slope parameters: $\beta_i \sim N(0, \text{prec} = 0.0001)$, $i = 1, 2, \ldots, p$

Prior for precision parameters: $\tau \sim \text{gamma}(1, 0.00005)$

Model 3:	All MH Outcomes	(Intercept)	-4.03	(-4.55, -3.52)
1st-Order		% Private Insurance	0.000628	(-0.00428, 0.00555)
random walk		% Public Insurance	0.0116	(0.00685, 0.0164)
		Median Age	0.0134	(0.00674, 0.0200)
		Median Income	-0.00490	(-0.0113, 0.00152)
		Education (% HS+)	0.00722	(0.0021, 0.0124)

Table S4: Poisson model with alternate prior(s) applied to all mental health outcomes

Prior for the intercept: $\beta_0 \sim N(0, \text{prec} = 0.00001)$

Prior for the slope parameters: $\beta_i \sim N(0, \text{prec} = 0.001)$, $i = 1, 2, \ldots, p$

Prior for precision parameters: $\tau \sim \text{gamma}(0.1, 0.00001)$

Model 3:	All MH Outcomes	(Intercept)	-4.03	(-4.55, -3.52)
1st-Order		% Private Insurance	0.000629	(-0.00428, 0.00555)
random walk		% Public Insurance	0.0116	(0.00685, 0.0164)
		Median Age	0.0134	(0.00674, 0.0200)
		Median Income	-0.00490	(-0.0113, 0.00152)
		Education (% HS+)	0.00722	(0.00210, 0.0124)

Table S5: Poisson model with alternate prior(s) applied to all mental health outcomes

Prior for the intercept: $\beta_0 \sim N(0, \text{prec} = 0)$

Prior for the slope parameters: $\beta_i \sim N(0, \text{prec} = 0.0001)$, $i = 1, 2, \ldots, p$

Prior for precision parameters: $\tau \sim \text{gamma}(0.1, 0.00001)$

Model 3:	All MH Outcomes	(Intercept)	-4.05	(-4.58, -3.52)
1st-Order		% Private Insurance	0.000718	(-0.00420, 0.00565)
random walk		% Public Insurance	0.0117	(0.00688, 0.0165)
		Median Age	0.0133	(0.00672, 0.0200)
		Median Income	-0.00474	(-0.0112, 0.00170)
		Education (% HS+)	0.007228	(0.00215, 0.0124)

Table S6: Poisson model with alternate prior(s) applied to all mental health outcomes

Prior for the intercept: $\beta_0 \sim N(0, \text{prec} = 0.00001)$

Prior for the slope parameters: $\beta_i \sim N(0, \text{prec} = 0.0001)$, $i = 1, 2, \ldots, p$

Prior for precision parameters: $\tau \sim \text{gamma}(1, 0.00005)$

Model 3:	All MH Outcomes	(Intercept)	-4.03	(-4.55, -3.52)
1st-Order		% Private Insurance	0.000628	(-0.00427, 0.00555)
random walk		% Public Insurance	0.0116	(0.00685, 0.0164)
		Median Age	0.0134	(0.00674, 0.0200)
		Median Income	-0.00490	(-0.0113, 0.00151)
		Education (% HS+)	0.00722	(0.00210, 0.0124)

Table S7: Poisson model with alternate prior(s) applied to all mental health outcomes

Prior for the intercept: $\beta_0 \sim N(0, \text{prec} = 0.00001)$

Prior for the slope parameters: $\beta_i \sim N(0, \text{prec} = 0.0001), i = 1, 2, \ldots, p$

Prior for precision parameters: $\tau \sim \text{gamma}(0.1, 0.00001)$

Model 3:	All MH Outcomes	(Intercept)	-4.05	(-4.57, -3.53)
1st-Order		% Private Insurance	0.000717	(-0.00420, 0.00565)
random walk		% Public Insurance	0.0117	(0.00688, 0.0165)
		Median Age	0.0133	(0.00672, 0.0200)
		Median Income	-0.00474	(-0.0112, 0.00169)
		Education (% HS+)	0.00728	(0.00215, 0.0124)

Table S8: Poisson model with alternate prior(s) applied to all mental health outcomes

2. Regression Analysis for Poisson Models

Bipolar Disorder $n = 5,524$

Model	Outcome	Parameter	Estimate & Credible Interval	
Model 1: Indicator variable for each year	Bipolar Disorder	(Intercept)	-5.52	(-6.52, -4.52)
		Year 2019	0.145	(0.0533, 0.237)
		Year 2020	-0.101	(-0.205, 0.00375)
		Year 2021	-0.0419	(-0.155, 0.0711)
		% Private Insurance	-0.00485	(-0.0151, 0.00553)
		% Public Insurance	0.0219	(0.0112, 0.0326)
		Median Age	0.0144	(0.000474, 0.0284)
		Median Income	-0.0153	(-0.0289, -0.00162)
		Education (% HS+)	0.00349	(-0.00792, 0.0149)
Model 2: Indicator variable for COVID	Bipolar Disorder	(Intercept)	-5.74	(-6.75, -4.75)
		COVID Indicator	-0.164	(-0.241, -0.0868)
		% Private Insurance	-0.00391	(-0.0141, 0.00643)
		% Public Insurance	0.024	(0.0134, 0.0347)
		Median Age	0.016	(0.00201, 0.03)
		Median Income	-0.00957	(-0.0227, 0.00354)
		Education (% HS+)	0.00278	(-0.00862, 0.0142)
Model 3: 1st-Order random walk	Bipolar Disorder	(Intercept)	-5.45	(-6.46, -4.44)
		% Private Insurance	-0.00468	(-0.0149, 0.00567)
		% Public Insurance	0.0216	(0.011, 0.0323)
		Median Age	0.0139	(-0.0000634, 0.0278)
		Median Income	-0.0166	(-0.0299, -0.00332)
		Education (% HS+)	0.00334	(-0.00804, 0.0147)

Table S9: Ecological spatial models for the three spatio-temporal models applied to bipolar disorder cases.

OCD $n = 118$

Model	Outcome	Parameter	Estimate &	Credible Interval
Model 1: Indicator variable for each year	OCD	(Intercept)	-9.34	(-13.3, -5.55)
		Year 2019	0.299	(-0.209, 0.806)
		Year 2020	0.143	(-0.39, 0.677)
		Year 2021	-0.264	(-0.876, 0.348)
		% Private Insurance	-0.0166	(-0.056, 0.0223)
		% Public Insurance	0.000806	(-0.0459, 0.0478)
		Median Age	0.0138	(-0.0468, 0.0746)
		Median Income	0.0208	(-0.0374, 0.0793)
		Education (% HS+)	0.014	(-0.0395, 0.0688)
Model 2: Indicator variable for COVID	OCD	(Intercept)	-9.24	(-13.1, -5.47)
		COVID Indicator	-0.2	(-0.588, 0.188)
		% Private Insurance	-0.0146	(-0.0536, 0.0241)
		% Public Insurance	0.00123	(-0.0449, 0.0476)
		Median Age	0.0149	(-0.0453, 0.0751)
		Median Income	0.0197	(-0.0363, 0.0759)
		Education (% HS+)	0.0127	(-0.04, 0.0667)
Model 3: 1st-Order random walk	OCD	(Intercept)	-9.1	(-13, -5.36)
		% Private Insurance	-0.0134	(-0.0522, 0.0251)
		% Public Insurance	-0.00122	(-0.0473, 0.045)
		Median Age	0.0132	(-0.0468, 0.0732)
		Median Income	0.0104	(-0.0423, 0.063)
		Education (% HS+)	0.0146	(-0.0374, 0.0681)

Table S10: Ecological spatial models for the three spatio-temporal models applied to OCD cases.

PTSD $n = 925$

Model	Outcome	Parameter	Estimate & Credible Interval	
Model 1: Indicator variable for each year	PTSD	(Intercept)	-5.71	(-7.37, -4.07)
		Year 2019	0.369	(0.189, 0.548)
		Year 2020	0.221	(0.0276, 0.415)
		Year 2021	-0.0522	(-0.271, 0.167)
		% Private Insurance	-0.00937	(-0.0258, 0.00722)
		% Public Insurance	0.000334	(-0.0186, 0.0194)
		Median Age	0.0303	(0.00516, 0.0555)
		Median Income	-0.00000942	(-0.0000332, 0.0000146)
		Education (% HS+)	-0.0127	(-0.0339, 0.00825)
Model 2: Indicator variable for COVID	PTSD	(Intercept)	-5.92	(-7.58, -4.3)
		COVID Indicator	-0.106	(-0.248, 0.0365)
		% Private Insurance	-0.00519	(-0.0214, 0.0113)
		% Public Insurance	0.0034	(-0.0153, 0.0223)
		Median Age	0.0326	(0.00781, 0.0577)
		Median Income	-0.00978	(-0.0327, 0.0133)
		Education (% HS+)	-0.0131	(-0.0338, 0.00748)
Model 3: 1st-Order random walk	PTSD	(Intercept)	-5.59	(-7.27, -3.94)
		% Private Insurance	-0.00817	(-0.0245, 0.00842)
		% Public Insurance	0.00044	(-0.0184, 0.0194)
		Median Age	0.0304	(0.00542, 0.0555)
		Median Income	-0.0116	(-0.0348, 0.0119)
		Education (% HS+)	-0.0126	(-0.0336, 0.00822)

Table S11: Ecological spatial models for the three spatio-temporal models applied to PTSD cases.

Psychosis $n = 2,491$

Model	Outcome	Parameter	Estimate & Credible Interval	
Model 1: Indicator variable for each year	Psychosis	(Intercept)	-3.85	(-5.07, -2.64)
		Year 2019	0.128	(0.016, 0.24)
		Year 2020	0.0077	(-0.116, 0.131)
		Year 2021	0.153	(0.0203, 0.287)
		% Private Insurance	-0.0105	(-0.0227, 0.00188)
		% Public Insurance	0.00365	(-0.00943, 0.0167)
		Median Age	0.00302	(-0.0145, 0.0205)
		Median Income	-0.0371	(-0.0541, -0.0202)
		Education (% HS+)	0.000538	(-0.0137, 0.0148)
Model 2: Indicator variable for COVID	Psychosis	(Intercept)	-4.12	(-5.33, -2.91)
		COVID Indicator	-0.00635	(-0.0976, 0.0848)
		% Private Insurance	-0.0101	(-0.0222, 0.00221)
		% Public Insurance	0.00577	(-0.00728, 0.0188)
		Median Age	0.00498	(-0.0125, 0.0225)
		Median Income	-0.029	(-0.0453, -0.0128)
		Education (% HS+)	-0.00043	(-0.0147, 0.0138)
Model 3: 1st-Order random walk	Psychosis	(Intercept)	-4.05	(-5.22, -2.88)
		% Private Insurance	-0.0102	(-0.0224, 0.00207)
		% Public Insurance	0.00533	(-0.00757, 0.0182)
		Median Age	0.00463	(-0.0129, 0.0221)
		Median Income	-0.0304	(-0.0461, -0.0148)
		Education (% HS+)	-0.000374	(-0.0146, 0.0138)

Table S12: Ecological spatial models for the three spatio-temporal models applied to psychosis cases.

Other Mental Health Diagnoses$n = 2,711$

Model	Outcome	Parameter	Estimate & Credible Interval	
Model 1: Indicator variable for each year	Other	(Intercept)	-4.35	(-5.32, -3.38)
		Year 2019	0.124	(0.0189, 0.229)
		Year 2020	-0.0416	(-0.156, 0.0725)
		Year 2021	0.00514	(-0.117, 0.127)
		% Private Insurance	-0.0145	(-0.0243, -0.00446)
		% Public Insurance	0.00246	(-0.00846, 0.0134)
		Median Age	0.0194	(0.00478, 0.0342)
		Median Income	-0.0112	(-0.0253, 0.00294)
		Education (% HS+)	-0.0025	(-0.0148, 0.00979)
Model 2: Indicator variable for COVID	Other	(Intercept)	-4.44	(-5.43, -3.47)
		COVID Indicator	-0.091	(-0.175, -0.00709)
		% Private Insurance	-0.0139	(-0.0237, -0.00396)
		% Public Insurance	0.00365	(-0.00728, 0.0146)
		Median Age	0.021	(0.00631, 0.0357)
		Median Income	-0.00755	(-0.0212, 0.00622)
		Education (% HS+)	-0.0037	(-0.016, 0.00859)
Model 3: 1st-Order random walk	Other	(Intercept)	-4.27	(-5.23, -3.32)
		% Private Insurance	-0.0141	(-0.0238, -0.00419)
		% Public Insurance	0.00219	(-0.00859, 0.013)
		Median Age	0.0195	(0.005, 0.0341)
		Median Income	-0.0121	(-0.0251, 0.000987)
		Education (% HS+)	-0.0032	(-0.0155, 0.00901)

Table S13: Ecological spatial models for the three spatio-temporal models applied to other cases.

2. Negative Binomial Models

All Mental Health Outcomes $n = 22,565$

Model	Outcome	Parameter	Estimate & Credible Interval	
Model 1: Indicator variable for each year	All MH Outcomes	(Intercept)	-3.54	(-4.54, -2.55)
		Year 2019	0.0852	(-0.0225, 0.193)
		Year 2020	-0.168	(-0.282, -0.0532)
		Year 2021	-0.304	(-0.428, -0.179)
		% Private Insurance	-0.0179	(-0.0278, -0.00789)
		% Public Insurance	0.00213	(-0.00903, 0.0133)
		Median Age	0.0234	(0.00889, 0.0381)
		Median Income	-0.00689	(-0.0206, 0.00689)
		Education (% HS+)	0.00326	(-0.00892, 0.0155)
Model 2: Indicator variable for COVID	All MH Outcomes	(Intercept)	-3.54	(-4.55, -2.55)
		COVID Indicator	-0.274	(-0.358, -0.188)
		% Private Insurance	-0.0166	(-0.0266, -0.00658)
		% Public Insurance	0.00246	(-0.00874, 0.0136)
		Median Age	0.0242	(0.00959, 0.0389)
		Median Income	-0.00818	(-0.00215, 0.00516)
		Education (% HS+)	0.00279	(-0.00945, 0.015)
Model 3: 1st-Order random walk	All MH Outcomes	(Intercept)	-3.60	(-4.61, -2.60)
		% Private Insurance	-0.0176	(-0.0275, -0.00766)
		% Public Insurance	0.00195	(-0.0092, 0.0131)
		Median Age	0.0231	(0.00862, 0.0377)
		Median Income	-0.00800	(-0.0217, 0.00572)
		Education (% HS+)	0.00323	(-0.00895, 0.0154)

Table S14: Regression results with a negative binomial model for counts when the outcome is all mental health diagnoses.

Anxiety $n = 7,255$

Model	Outcome	Parameter	Estimate & Credible Interval	
Model 1: Indicator variable for each year	Anxiety	(Intercept)	-4.18	(-4.90, -3.48)
		Year 2019	0.0884	(0.025, 0.152)
		Year 2020	-0.109	(-0.179, -0.038)
		Year 2021	-0.253	(-0.333, -0.173)
		% Private Insurance	-0.0138	(-0.0209, -0.00669)
		% Public Insurance	0.00647	(-0.00118, 0.0141)
		Median Age	0.0101	(-0.0000158, 0.0203)
		Median Income	-0.00761	(-0.0198, 0.00464)
		Education (% HS+)	0.0147	(0.00647, 0.023)
Model 2: Indicator variable for COVID	Anxiety	(Intercept)	-3.89	(-4.76, -3.02)
		COVID Indicator	-0.208	(-0.285, -0.131)
		% Private Insurance	-0.0172	(-0.026, -0.00837)
		% Public Insurance	0.00475	(-0.00516, 0.0147)
		Median Age	0.0135	(0.000301, 0.0267)
		Median Income	-0.0086	(-0.0205, 0.00336)
		Education (% HS+)	0.0120	(0.000899, 0.0232)
Model 3: 1st-Order random walk	Anxiety	(Intercept)	-3.90	(-4.78, -3.03)
		% Private Insurance	-0.0180	(-0.0267, -0.00927)
		% Public Insurance	0.00415	(-0.00572, 0.0140)
		Median Age	0.0125	(-0.000632, 0.0256)
		Median Income	-0.00868	(-0.0209, 0.00353)
		Education (% HS+)	0.0124	(0.00141, 0.0235)

Table S15: Regression results with a negative binomial model for counts when the outcome is anxiety.

Bipolar Disorder $n = 3,524$

Model	Outcome	Parameter	Estimate & Credible Interval	
Model 1: Indicator variable for each year	Bipolar Disorder	(Intercept)	-4.61	(-5.96, -3.27)
		Year 2019	0.133	(-0.0293, 0.296)
		Year 2020	-0.0871	(-0.258, 0.0842)
		Year 2021	-0.0191	(-0.199, 0.161)
		% Private Insurance	-0.0172	(-0.0309, -0.00338)
		% Public Insurance	0.0129	(-0.00263, 0.0284)
		Median Age	0.0194	(-0.000684, 0.0394)
		Median Income	-0.00843	(-0.0275, 0.0107)
		Education (% HS+)	-0.000187	(-0.0177, 0.0173)
Model 2: Indicator variable for COVID	Bipolar Disorder	(Intercept)	-4.67	(-6.03, -3.32)
		COVID Indicator	-0.131	(-0.255, -0.00672)
		% Private Insurance	-0.0171	(-0.0309, -0.00324)
		% Public Insurance	0.0142	(-0.00132, 0.0297)
		Median Age	0.0202	(0.000153, 0.0403)
		Median Income	-0.00474	(-0.0234, 0.0140)
		Education (% HS+)	-0.00114	(-0.0187, 0.0163)
Model 3: 1st-Order random walk	Bipolar Disorder	(Intercept)	-4.53	(-5.87, -3.20)
		% Private Insurance	-0.0169	(-0.0306, -0.00310)
		% Public Insurance	0.0127	(-0.00267, 0.0282)
		Median Age	0.0184	(-0.00162, 0.0384)
		Median Income	-0.0101	(-0.0281, 0.00794)
		Education (% HS+)	-0.0000471	(-0.0176, 0.0175)

Table S16: Regression results with a negative binomial model for counts when the outcome is bipolar.

Depression $n = 5,298$

Model	Outcome	Parameter	Estimate & Credible Interval	
Model 1: Indicator variable for each year	Depression	(Intercept)	-8.01	(-11.2, -4.88)
		Year 2019	0.245	(-0.385, 0.870)
		Year 2020	0.122	(-0.525, 0.765)
		Year 2021	-0.251	(-0.959, 0.457)
		% Private Insurance	-0.00927	(-0.0415, 0.0231)
		% Public Insurance	-0.00180	(-0.0423, 0.0389)
		Median Age	0.0144	(-0.0380, 0.0670)
		Median Income	-0.0034	(-0.0168, 0.0100)
		Education (% HS+)	-0.00530	(-0.0501, 0.0394)
Model 2: Indicator variable for COVID	Depression	(Intercept)	-4.28	(-5.25, -3.32)
		COVID Indicator	-0.383	(-0.465, -0.301)
		% Private Insurance	-0.0143	(-0.0240, -0.00470)
		% Public Insurance	0.00608	(-0.00475, 0.0169)
		Median Age	0.0223	(0.00815, 0.0366)
		Median Income	-0.0074	(-0.0204, 0.00565)
		Education (% HS+)	0.00517	(-0.00674, 0.0171)
Model 3: 1st-Order random walk	Depression	(Intercept)	-4.48	(-5.46, -3.52)
		% Private Insurance	-0.0155	(-0.0250, -0.00597)
		% Public Insurance	0.00644	(-0.00429, 0.0171)
		Median Age	0.0214	(0.00733, 0.0355)
		Median Income	-0.00427	(-0.0176, 0.00912)
		Education (% HS+)	0.00535	(-0.00641, 0.0171)

Table S17: Regression results with a negative binomial model for counts when the outcome is depression.

OCD $n = 118$

Model	Outcome	Parameter	Estimate & Credible Interval	
Model 1: Indicator variable for each year	OCD	(Intercept)	-8.01	(-11.2, -4.88)
		Year 2019	0.245	(-0.385, 0.870)
		Year 2020	0.122	(-0.525, 0.765)
		Year 2021	-0.251	(-0.959, 0.457)
		% Private Insurance	-0.00927	(-0.0415, 0.0231)
		% Public Insurance	-0.00180	(-0.0423, 0.0389)
		Median Age	0.0144	(-0.0380, 0.0670)
		Median Income	0.0166	(-0.0334, 0.0665)
		Education (% HS+)	-0.00530	(-0.0501, 0.0394)
Model 2: Indicator variable for COVID	OCD	(Intercept)	-7.99	(-11.1, -4.87)
		COVID Indicator	-0.176	(-0.638, 0.287)
		% Private Insurance	-0.00755	(-0.0396, 0.0247)
		% Public Insurance	-0.00143	(-0.0418, 0.0392)
		Median Age	0.0160	(-0.0361, 0.0683)
		Median Income	0.0146	(-0.0344, 0.0635)
		Education (% HS+)	-0.00533	(-0.0497, 0.0390)
Model 3: 1st-Order random walk	OCD	(Intercept)	-7.94	(-11.1, -4.83)
		% Private Insurance	-0.00714	(-0.0391, 0.0250)
		% Public Insurance	-0.00278	(-0.0429, 0.0376)
		Median Age	0.0150	(-0.0368, 0.0670)
		Median Income	0.0108	(-0.0368, 0.0583)
		Education (% HS+)	-0.00472	(-0.0488, 0.0393)

Table S18: Regression results with a negative binomial model for counts when the outcome is OCD.

PTSD $n = 925$

Model	Outcome	Parameter	Estimate & Credible Interval	
Model 1: Indicator variable for each year	PTSD	(Intercept)	-5.06	(-6.97, -3.18)
		Year 2019	0.391	(0.134, 0.649)
		Year 2020	0.189	(-0.0769, 0.456)
		Year 2021	-0.0327	(-0.323, 0.258)
		% Private Insurance	-0.0161	(-0.0353, 0.00316)
		% Public Insurance	-0.00621	(-0.0289, 0.0167)
		Median Age	0.0318	(0.00203, 0.0620)
		Median Income	-0.0101	(-0.0338, 0.0139)
		Education (% HS+)	-0.0136	(-0.0404, 0.0129)
Model 2: Indicator variable for COVID	PTSD	(Intercept)	-5.09	(-7.02, -3.19)
		COVID Indicator	-0.126	(-0.322, 0.0699)
		% Private Insurance	-0.0135	(-0.0328, 0.00587)
		% Public Insurance	-0.00401	(-0.0268, 0.0190)
		Median Age	0.0340	(0.00405, 0.0644)
		Median Income	-0.0083	(-0.0364, 0.0203)
		Education (% HS+)	-0.0156	(-0.0425, 0.0111)
Model 3: 1st-Order random walk	PTSD	(Intercept)	-5.01	(-6.91, -3.12)
		% Private Insurance	-0.0133	(-0.0325, 0.00596)
		% Public Insurance	-0.00512	(-0.0278, 0.0177)
		Median Age	0.0322	(0.00246, 0.0622)
		Median Income	-0.0127	(-0.0398, 0.0147)
		Education (% HS+)	-0.0143	(-0.0410, 0.0122)

Table S19: Regression results with a negative binomial model for counts when the outcome is PTSD.

Psychosis $n = 2,491$

Model	Outcome	Parameter	Estimate & Credible Interval	
Model 1: Indicator variable for each year	Psychosis	(Intercept)	-3.35	(-5.12, -1.57)
		Year 2019	0.0911	(-0.125, 0.307)
		Year 2020	-0.0278	(-0.254, 0.198)
		Year 2021	0.106	(-0.129, 0.340)
		% Private Insurance	-0.0273	(-0.0456, -0.00886)
		% Public Insurance	-0.00036	(-0.0207, 0.0199)
		Median Age	0.00840	(-0.0186, 0.0354)
		Median Income	-0.032	(-0.0576, -0.00653)
		Education (% HS+)	0.00547	(-0.0177, 0.0285)
Model 2: Indicator variable for COVID	Psychosis	(Intercept)	-3.42	(-5.19, -1.65)
		COVID Indicator	-0.0140	(-0.178, 0.150)
		% Private Insurance	-0.0274	(-0.0456, -0.00896)
		% Public Insurance	0.000805	(-0.0195, 0.0210)
		Median Age	0.00930	(-0.0176, 0.0362)
		Median Income	-0.0283	(-0.0533, -0.00342)
		Education (% HS+)	0.00464	(-0.0184, 0.0276)
Model 3: 1st-Order random walk	Psychosis	(Intercept)	-3.40	(-5.16, -1.64)
		% Private Insurance	-0.0273	(-0.0455, -0.00892)
		% Public Insurance	0.000636	(-0.0195, 0.0208)
		Median Age	0.00911	(-0.0177, 0.0359)
		Median Income	-0.0290	(-0.0532, -0.00483)
		Education (% HS+)	0.00468	(-0.0183, 0.0276)

Table S20: Regression results with a negative binomial model for counts when the outcome is psychosis.

Substance Abuse $n = 243$

Model	Outcome	Parameter	Estimate & Credible Interval	
Model 1: Indicator variable for each year	Substance Abuse	(Intercept)	-6.18	(-8.01, -4.32)
		Year 2019	0.208	(-0.187, 0.603)
		Year 2020	-0.0266	(-0.445, 0.392)
		Year 2021	-0.355	(-0.819, 0.107)
		% Private Insurance	-0.0240	(-0.0431, -0.00496)
		% Public Insurance	0.0128	(-0.00933, 0.0351)
		Median Age	0.00268	(-0.0315, 0.0364)
		Median Income	-0.0231	(-0.0576, 0.0114)
		Education (% HS+)	0.00639	(-0.0253, 0.0381)
Model 2: Indicator variable for COVID	Substance Abuse	(Intercept)	-6.19	(-8.03, -4.35)
		COVID Indicator	-0.277	(-0.584, 0.0277)
		% Private Insurance	-0.0224	(-0.0413, -0.00341)
		% Public Insurance	0.0135	(-0.00850, 0.0357)
		Median Age	0.00438	(-0.0296, 0.0378)
		Median Income	-0.0243	(-0.0581, 0.00944)
		Education (% HS+)	0.00596	(-0.0255, 0.0374)
Model 3: 1st-Order random walk	Substance Abuse	(Intercept)	-6.17	(-8.00, -4.34)
		% Private Insurance	-0.0220	(-0.0409, -0.00316)
		% Public Insurance	0.0122	(-0.00970, 0.0343)
		Median Age	0.00299	(-0.0307, 0.0361)
		Median Income	-0.0298	(-0.0624, 0.00281)
		Education (% HS+)	0.00738	(-0.0235, 0.0384)

Table S21: Regression results with a negative binomial model for counts when the outcome is substance abuse.

Other $n = 2,711$

Model	Outcome	Parameter	Estimate & Credible Interval	
Model 1: Indicator variable for each year	Other	(Intercept)	-3.83	(-4.98, -2.68)
		Year 2019	0.130	(-0.0321, 0.293)
		Year 2020	0.00825	(-0.161, 0.179)
		Year 2021	0.0485	(-0.128, 0.226)
		% Private Insurance	-0.0206	(-0.0322, -0.00889)
		% Public Insurance	-0.00268	(-0.0164, 0.0109)
		Median Age	0.0196	(0.00176, 0.0375)
		Median Income	-0.0168	(-0.034, 0.000573)
		Education (% HS+)	0.000112	(-0.0157, 0.0159)
Model 2: Indicator variable for COVID	Other	(Intercept)	-3.84	(-4.99, -2.69)
		COVID Indicator	-0.0443	(-0.167, 0.0788)
		% Private Insurance	-0.0205	(-0.0321, -0.00881)
		% Public Insurance	-0.0190	(-0.0156, 0.0117)
		Median Age	0.0204	(0.00261, 0.0385)
		Median Income	-0.0142	(-0.0312, 0.00282)
		Education (% HS+)	-0.000803	(-0.0167, 0.0150)
Model 3: 1st-Order random walk	Other	(Intercept)	-3.80	(-4.94, -2.66)
		% Private Insurance	-0.0205	(-0.0321, -0.00885)
		% Public Insurance	-0.00239	(-0.0160, 0.0111)
		Median Age	0.0198	(0.00207, 0.0376)
		Median Income	-0.0159	(-0.0323, 0.000577)
		Education (% HS+)	-0.000337	(-0.0161, 0.0154)

Table S22: Regression results with a negative binomial model for counts when the outcome is all other mental health diagnoses.
